# Hippo–YAP/TAZ signalling coordinates adipose plasticity and energy balance by uncoupling leptin expression from fat mass

**DOI:** 10.1038/s42255-024-01045-4

**Published:** 2024-05-29

**Authors:** Sungwoo Choi, Ju-Gyeong Kang, Yen T. H. Tran, Sun-Hye Jeong, Kun-Young Park, Hyemi Shin, Young Hoon Kim, Myungsun Park, Hahn Nahmgoong, Taejun Seol, Haeyon Jeon, Yeongmin Kim, Sanghee Park, Hee-joo Kim, Min-Seob Kim, Xiaoxu Li, Maroun Bou Sleiman, Eries Lee, Jinhyuk Choi, David Eisenbarth, Sang Heon Lee, Suhyeon Cho, David D. Moore, Johan Auwerx, Il-Young Kim, Jae Bum Kim, Jong-Eun Park, Dae-Sik Lim, Jae Myoung Suh

**Affiliations:** 1 Graduate School of Medical Science and Engineering, Korea Advanced Institute of Science and Technology, Daejeon, Republic of Korea; 2 National Creative Research Center for Cell Plasticity, KAIST Stem Cell Center, Department of Biological Sciences, Korea Advanced Institute of Science and Technology, Daejeon, Republic of Korea; 3 National Creative Research Initiatives Center for Adipocyte Structure and Function, Institute of Molecular Biology and Genetics, School of Biological Sciences, Seoul National University, Seoul, Republic of Korea; 4 Department of Health Sciences and Technology, Gachon Advanced Institute for Health Sciences & Technology, Gachon University, Incheon, Republic of Korea; 5 Department of Molecular Medicine, Lee Gil Ya Cancer and Diabetes Institute, College of Medicine, Gachon University, Incheon, Republic of Korea; 6 Department of Fundamental Environment Research, Environmental Measurement and Analysis Center, National Institute of Environmental Research, Incheon, Republic of Korea; 7 Laboratory of Integrative Systems Physiology, École Polytechnique Fédérale de Lausanne (EPFL), Lausanne, Switzerland; 8 Department of Nutritional Sciences and Toxicology, University of California, Berkeley, Berkeley, CA USA

**Keywords:** Obesity, Cell signalling, Fat metabolism

## Abstract

Adipose tissues serve as an energy reservoir and endocrine organ, yet the mechanisms that coordinate these functions remain elusive. Here, we show that the transcriptional coregulators, YAP and TAZ, uncouple fat mass from leptin levels and regulate adipocyte plasticity to maintain metabolic homeostasis. Activating YAP/TAZ signalling in adipocytes by deletion of the upstream regulators *Lats1* and *Lats2* results in a profound reduction in fat mass by converting mature adipocytes into delipidated progenitor-like cells, but does not cause lipodystrophy-related metabolic dysfunction, due to a paradoxical increase in circulating leptin levels. Mechanistically, we demonstrate that YAP/TAZ–TEAD signalling upregulates leptin expression by directly binding to an upstream enhancer site of the leptin gene. We further show that YAP/TAZ activity is associated with, and functionally required for, leptin regulation during fasting and refeeding. These results suggest that adipocyte Hippo–YAP/TAZ signalling constitutes a nexus for coordinating adipose tissue lipid storage capacity and systemic energy balance through the regulation of adipocyte plasticity and leptin gene transcription.

## Abstract (web summary)

The transcriptional coregulators YAP/TAZ are shown to directly control leptin gene transcription, thereby uncoupling adipose tissue mass from leptin levels.

## Main

Adipose tissue is a metabolic and endocrine organ that regulates systemic energy balance^1^. White fat depots store excess energy in the form of triglycerides, while they also secrete adipokines such as leptin and adiponectin to control systemic energy expenditure and food intake^1,2^. The energy storage and endocrine functions of adipose tissue both contribute to the proper regulation of systemic energy homeostasis, with defects in either function resulting in metabolic disease^2,3^. In obesity-associated metabolic syndrome, excess energy that cannot be stored in adipose tissue spills over to peripheral organs in the form of fatty acids, resulting in fatty liver, hyperglycaemia and insulin resistance^2,3^. Defects in energy storage and endocrine functions caused by lipodystrophy characterized by partial or total adipose tissue loss also result in a severe diabetic phenotype^4,5^. Of note, restoring adipose endocrine function via leptin replacement has been shown to reverse lipodystrophy-associated metabolic dysfunction^6,7^. While it is widely accepted that circulating leptin levels are proportional to fat mass^8,9^, the molecular mechanism connecting fat mass and leptin expression remains poorly understood.

Hippo–YAP/TAZ signalling coordinates the control of organ size and cell-type-specific functions through cooperation with tissue-specific transcription factors^10,11^. YAP and TAZ are the downstream effectors of the Hippo signalling pathway, and their activity is determined by phosphorylation status^12^. The upstream kinases LATS1 and LATS2 directly phosphorylate YAP/TAZ, resulting in their cytoplasmic localization and degradation^12^. LATS1/LATS2 deficiency allows YAP/TAZ to translocate to the nucleus, where they act as transcriptional coactivators or corepressors^13,14^. With regard to adipocyte biology, TAZ inhibits adipogenesis by inhibiting peroxisome proliferator–activated receptor-γ (PPARG), an essential transcription factor for adipogenesis^15^. YAP/TAZ are inactivated during adipocyte differentiation and have thus been thought to be dispensable for mature adipocyte function^16^. However, the loss of TAZ in differentiated adipocytes was recently found to increase PPARG activity and reduce insulin resistance in mice with diet-induced obesity^17^. Another study showed that adipocyte YAP/TAZ are activated by high-fat diet feeding^18^, further suggesting a role for YAP/TAZ in mature adipocytes. Here, using adipocyte-specific *Lats1/Lats2* knockout mice, we sought to investigate the physiological role of Hippo–YAP/TAZ signalling in adipose tissue homeostasis and its impact on systemic metabolism.

## Results

### Adipose YAP/TAZ activation results in severe lipoatrophy

**Fig. 1:**
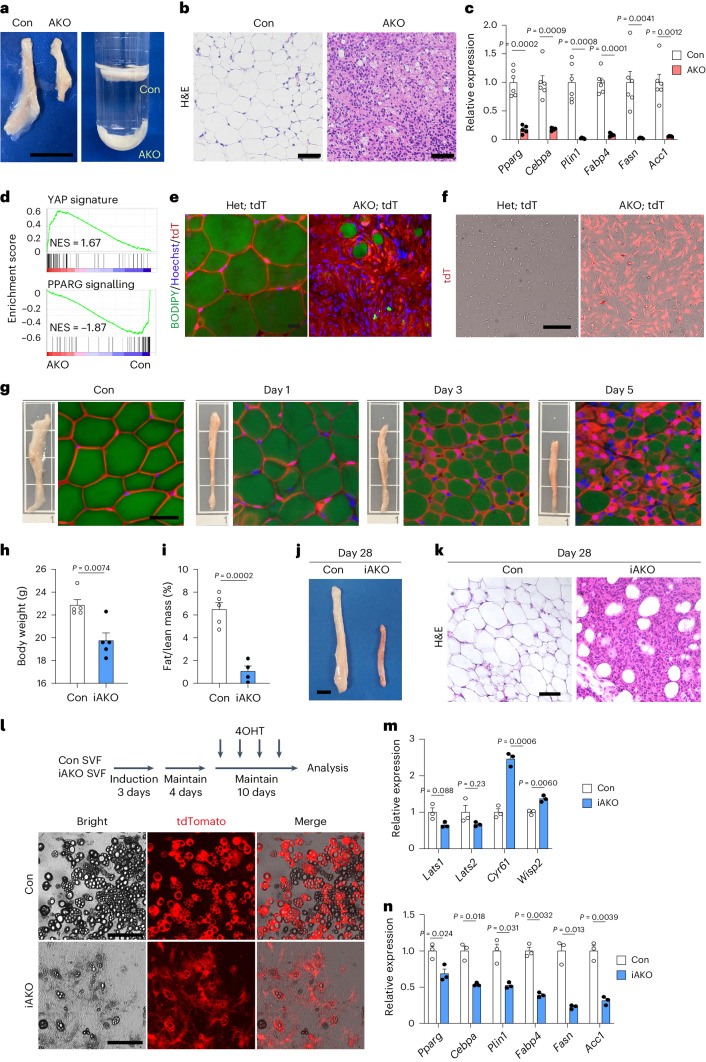
Adipose-specific *Lats1/Lats2* knockout mice develop lipoatrophy due to regression of mature adipocytes to progenitor-like cells. **a**–**d**, iWAT of 4-week-old *Lats1*^fl/fl^; *Lats2*^fl/fl^ (control (Con)) and *Adipoq-Cre; Lats1*^fl/fl^; *Lats2*^fl/fl^ (AKO) mice was analysed for gross morphology (scale bar, 1 cm) (**a**), histology (scale bar, 50 µm) (**b**), adipocyte gene expression (*n* = 7 Con, *n* = 5 AKO) (**c**) and enrichment score plots for YAP signature (top) and PPARG signalling (bottom) gene sets from RNA-seq analysis. NES, normalized enrichment score (*n* = 3 per genotype) (**d**). **e**, iWAT of 4-week-old *Adipoq-Cre; Lats1*^fl/+^; *Lats2*^fl/+^; *Rosa-LSL-tdTomato* (Het; tdT) and *Adipoq-Cre; Lats1*^fl/fl^; *Lats2*^fl/fl^; *Rosa-LSL-tdTomato* (AKO; tdT) mice was subjected to whole-mount fluorescence imaging for tdT expression (red) marking Cre recombinase activity, BODIPY staining (green) of lipid droplets and Hoechst 33342 staining (blue) of nuclei. Scale bar, 20 µm. **f**, tdT fluorescence (red) microscopy of the SVF isolated from iWAT in mice as in **e**. Scale bar, 100 µm. **g**, iWAT of 8- to 10-week-old *Lats1*^fl/fl^; *Lats2*^fl/fl^; *Rosa-LSL-tdTomato* (Con) and *Adipoq-CreER*^*T2*^; *Lats1*^fl/fl^; *Lats2*^fl/fl^; *Rosa-LSL-tdTomato* (iAKO) mice treated with tamoxifen and analysed for gross morphology and whole-mount fluorescence imaging of tdT expression (red), BODIPY (green) and Hoechst (blue) staining at 1, 3 or 5 days after the final tamoxifen treatment. Scale bar, 50 µm. **h**–**k**, Mice as in **g** were analysed for body weight (**h**), fat/lean mass ratio (**i**), gross morphology of iWAT (scale bar, 1 cm) (**j**) and histology of iWAT (scale bar, 50 µm) (**k**) at 28 days after the final tamoxifen injection (*n* = 5 per genotype). **l**, SVF isolated from iWAT of Con or iAKO mice at 6 to 8 weeks of age was treated with adipogenic induction media for 3 days, maintained for 4 days, treated with 4-hydroxytamoxifen (4OHT) for 10 days, and analysed by bright-field (Bright) and fluorescence (tdT) microscopy. Scale bar, 100 µm. **m**,**n**, RT–qPCR analysis of *Lats1*, *Lats2* and YAP/TAZ target gene expression (**m**) and adipocyte gene expression (**n**) in SVF-differentiated adipocytes from iWAT of mice as in **l** (*n* = 3 per genotype). Source data

**Extended Data Fig. 1:**
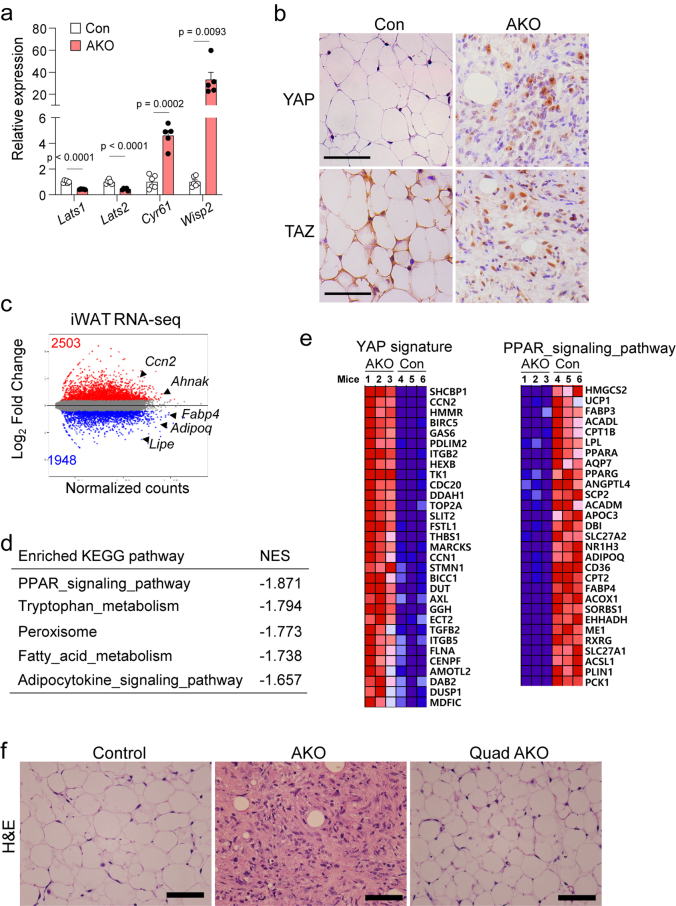
Adipocyte-specific deletion of Lats1/2 activates YAP/TAZ and attenuates PPAR signaling. **a**, RT-qPCR analysis of the expression of *Lats1*, *Lats2*, and YAP/TAZ target genes in iWAT of 4-week-old control (Con) and AKO male mice as in Fig. 1a–c. **b**, Immunohistochemical staining of YAP and TAZ in iWAT of control and AKO mice as in **a**. Scale bar = 50 μm. Data expressed as mean ± s.e.m. (n = 6 Con, n = 5 AKO). Data were analyzed by two-tailed unpaired t test. **c-e**, RNA-seq analysis was performed using total RNA from iWAT of 4-week-old *Lats1*^*fl/fl*^*; Lats2*^*fl/fl*^*; Rosa-LSL-tdTomato* (control, Con) or *Adipoq-Cre; Lats1*^*fl/fl*^*; Lats2*^*fl/fl*^*; Rosa-LSL-tdTomato* (AKO; tdT) male mice (n = 3 per genotype). MA plot of differentially expressed genes (DEGs) from iWAT RNA-seq data. Significantly changed (p < 0.05) genes were shown by red (fold change ≥ 2) or blue (fold change ≤ -2), with representative genes in arrow (**c**). GSEA analysis with KEGG pathway gene sets showing the top 5 significantly downregulated pathways (all p < 0.001) in AKO compared to control iWAT. NES, normalized enrichment score (**d**). Heatmaps to visualize DEGs related to the YAP signature (left, Cordenonsi et al. Cell 2011) and PPARG signaling pathways (right). Gene symbols represent upregulated (in red) or downregulated (in blue) core enrichment genes that contribute most to the enrichment result (**e**). **f**, Inguinal white adipose tisssue (iWAT) H&E histology of 4-week-old of *Lats1*^*fl/fl*^*; Lats2*^*fl/fl*^*; Yap*^*fl/fl*^*; Taz*^*fl/fl*^ (Control), *Adipoq-Cre; Lats1*^*fl/fl*^*; Lats2*^*fl/fl*^ (AKO), and *Adipoq-Cre; Lats1*^*fl/fl*^*; Lats2*^*fl/fl*^*; Yap*^*fl/fl*^*; Taz*^*fl/fl*^ (Quad AKO). Scale bar = 50 μm. Source data

To explore the function of Hippo–YAP/TAZ signalling in adipose tissues, we generated adipose-specific *Lats1* and *Lats2* knockout (AKO) mice by crossing mice homozygous for floxed alleles of *Lats1* and *Lats2* (*Lats1*^fl/fl^; *Lats2*^fl/fl^) with *Adipoq-Cre* mice expressing Cre recombinase under the control of the mouse adiponectin gene promoter. As expected, the expression of *Lats1* and *Lats2* was significantly reduced, while the expression of YAP/TAZ targets *Ccn1* (*Cyr61*) and *Ccn5* (*Wisp2*) was significantly increased in inguinal white adipose tissue (iWAT) of AKO mutant mice (Extended Data Fig. 1a). Additionally, a marked increase in the extent of nuclear localization of YAP and TAZ further confirmed the activated status of YAP/TAZ in iWAT of AKO mice (Extended Data Fig. 1b). When compared to control mice, AKO mice had markedly smaller iWAT, which, unlike its equivalent in control mice, did not float in phosphate-buffered saline (PBS; Fig. 1a). Histological examination revealed a striking loss of lipid droplet-bearing adipocytes in iWAT of AKO mice (Fig. 1b). Consistent with the lipoatrophic phenotype of AKO mice, the expression of adipocyte marker genes including *Pparg*, *Cebpa*, *Plin1*, *Fabp4*, *Fasn* and *Acaca* (*Acc1*) was significantly attenuated in iWAT of AKO mice compared to control (Fig. 1c). Gene-set enrichment analysis (GSEA) using oncogenic genes and the Kyoto Encyclopedia of Genes and Genomes (KEGG) pathway gene sets indicated that YAP signature genes^19^ were upregulated, while PPARG signalling genes were downregulated in the iWAT transcriptomes of AKO mice compared to control (Fig. 1d and Extended Data Fig. 1c–e). To confirm that the observed phenotype is due to YAP and TAZ activation, the canonical targets of LATS1 and LATS2, we generated adipose-specific *Lats1*, *Lats2*, *Yap1* (*Yap*) and *Wwtr1* (*Taz*) quadruple KO mice (Quad AKO). The Quad AKO mice showed a reversal of the lipoatrophy phenotype observed in AKO mice, indicating that YAP/TAZ function is essential for *Lats1* and *Lats2* deletion-induced lipoatrophy (Extended Data Fig. 1f). Together, these results demonstrate that adipose-specific deletion of *Lats1/Lats2* and consequent activation of YAP/TAZ severely impaired the maintenance of mature adipocytes.

**Extended Data Fig. 2:**
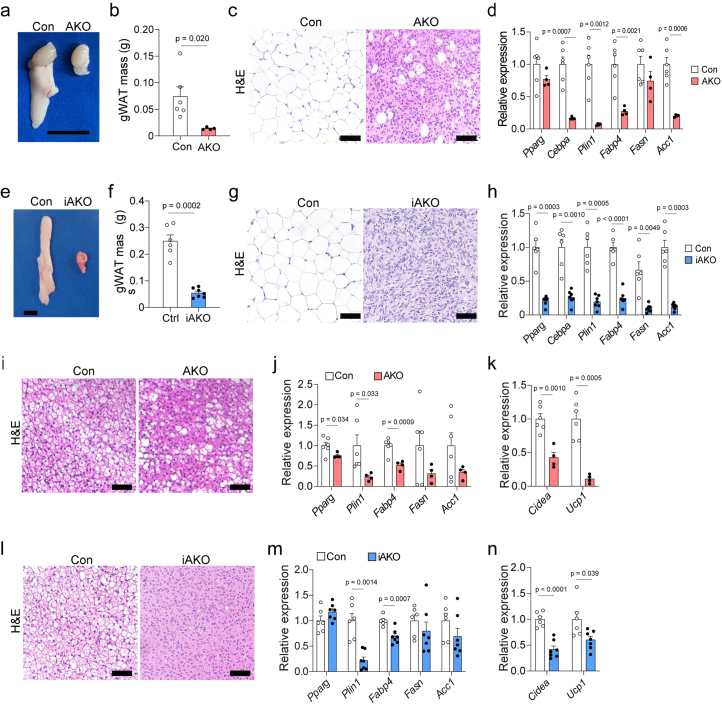
Adipocyte-specific deletion of Lats1/2 leads to loss of lipid content and decreased mass in gWAT and BAT. **a–d**, Gross morphology (**a**), tissue mass (**b**), H&E staining (scale bar = 50 μm) (**c**), and expression of mature adipocyte marker genes (**d**) for gWAT of 4-week-old control (Con) and AKO male mice as in Fig. 1a–c (n = 6 Con, n = 5 AKO). **e–h**, Gross morphology (**e**), tissue mass (**f**), H&E staining (scale bar = 50 μm) (**g**), and expression of mature adipocyte marker genes (**h**) for gWAT of 8- to 10-week-old control (Con) and iAKO male mice at 28 days after the final tamoxifen treatment as in Fig. 1g–k (n = 6 Con, n = 7 iAKO). **i–k**, H&E staining (scale bar = 50 μm) (**i**), and expression of mature adipocyte marker genes (**j**), and brown fat activation marker genes (**k**) for BAT of 4-week-old control (Con) and AKO male mice as in Fig. 1a–c (n = 6 Con, n = 5 AKO). **l–n**, H&E staining (scale bar = 50 μm) (**l**), and expression of mature adipocyte marker genes (**m**), and brown fat activation marker genes (**n**) for BAT of 8- to 10-week-old control (Con) and iAKO male mice at 28 days after the final tamoxifen treatment as in Fig. 1g–k (n = 6 Con, n = 7 iAKO). Data expressed as mean ± s.e.m. Data were analyzed by two-tailed unpaired t test. Source data

**Extended Data Fig. 3:**
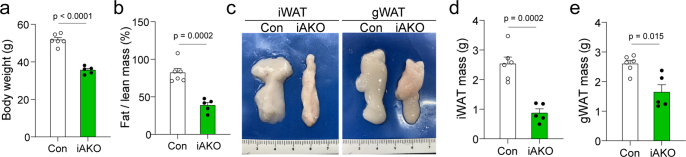
Inducible deletion of adipose Lats1/2 reduces fat mass in diet-induced obese mice. **a-e**, *Lats1*^*fl/fl*^; *Lats2*^*fl/fl*^ (Con) and *Adipoq-CreER*; *Lats1*^*fl/fl*^; *Lats2*^*fl/fl*^ (iAKO) male mice aged 8 to 10 weeks were fed high fat diet for 4 months, followed by three intraperitoneal injection of tamoxifen (100 mg/kg) every other day. Body weight (**a**), fat mass/lean mass ratio (**b**), gross morphology of iWAT and gWAT (**c**), iWAT mass (**d**), and gWAT mass (**e**) was assessed at 28 days after the final tamoxifen injection (n = 6 Con, n = 5 iAKO). Data expressed as mean ± s.e.m. Data were analzyed by two-tailed unpaired t test. Source data

**Extended Data Fig. 4:**
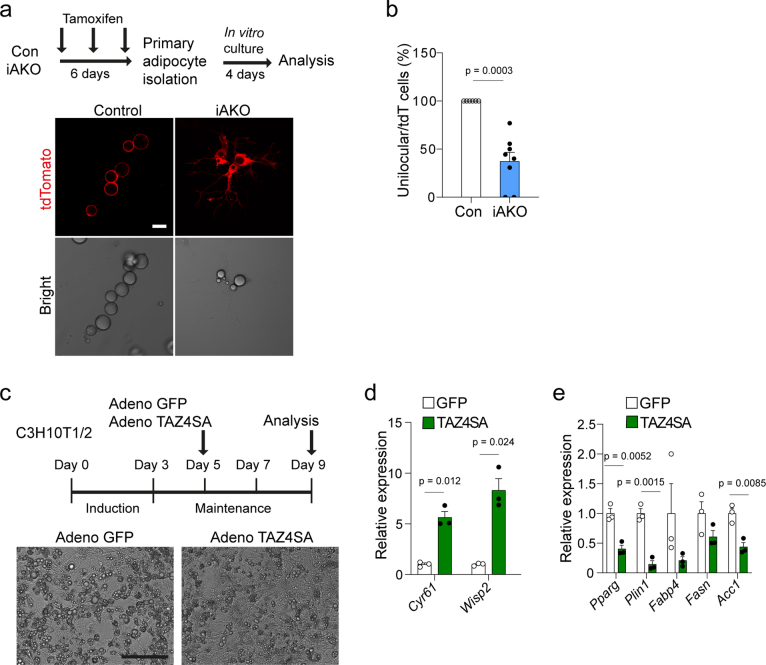
Inducible deletion of Lats1/2 or TAZ activation disrupts adipocyte maintenance *in vitro.* **a**, *Adipoq-CreER*; *Lats1*^*fl/+*^; *Lats2*^*fl/+*^; *Rosal-LSL-tdTomato* (Control, Con) and *Adipoq-CreER*; *Lats1*^*fl/fl*^; *Lats2*^*fl/fl*^; *Rosal-LSL-tdTomato* (iAKO; tdT) male mice at 8 to 10 weeks of age were treated with tamoxifen (100 mg/kg) every other day for three times (n = 3 Con, n = 3 iAKO). Primary adipocytes were isolated from iWAT of Con and iAKO; tdT mice at one day after the final tamoxifen injection, cultured for 4 days and then subjected to bright-field microscopy and fluorescence microscopy of tdTomato (tdT) expression (red). Scale bar = 50 μm. **b**, Proportion of unilocular cells among tdT+ cells for cultures as in **a** (n = 6 Con images, n = 8 iAKO images). **c**, C3H10T1/2 cells were exposed to inducers of adipogenesis for 3 days, maintained for 2 days, infected with adenoviruses encoding either a hyperactive form of TAZ (TAZ4SA) or green fluorescent protein (GFP) for 2 days, and then maintained in culture for an additional 2 days before observation by bright-field microscopy. Scale bar = 250 μm. **d, e**, RT-qPCR analysis of the expression of YAP/TAZ target genes (**d**) and of mature adipocyte marker genes (**e**) in cells treated as in **c** (n = 3 per condition). Data expressed as mean ± s.e.m. Data were analyzed by two-tailed unpaired t test. Source data

To explore the cause for the observed loss of mature adipocytes in the iWAT of AKO mice, we performed adipocyte lineage tracing. Analysis of mutant mice carrying the *Rosa-LSL-tdTomato* Cre reporter allele revealed that the majority of lipid-deficient cells in AKO mice iWAT were tdTomato positive (tdT^+^), indicating an adipocyte origin (Fig. 1e). These fibroblast-like tdT^+^ cells could be isolated from the stromal vascular fraction (SVF) of iWAT, cultured and passaged in vitro (Fig. 1f), suggesting that adipocyte-specific *Lats1/Lats2* deletion induces lipoatrophy as a result of the conversion of adipocytes to fibroblast-like cells. To further validate the effects of *Lats1/Lats2* deletion in fully differentiated adipocytes, we generated an inducible adipocyte-specific *Lats1/Lats2* knockout model using *Adipoq-CreER*^*T2*^ and *Rosa-LSL-tdTomato* alleles (iAKO mice). Tamoxifen-induced deletion of *Lats1/Lats2* in mature adipocytes of adult iAKO mice resulted in a rapid and progressive reduction of iWAT size (Fig. 1g). Adipocyte lineage tracing, utilizing the *Rosa-LSL-tdTomato* Cre reporter allele, confirmed a corresponding decrease in cell size occurs in mutant adipocytes resulting from tamoxifen-induced deletion of *Lats1/Lats2* (Fig. 1g). Similarly to AKO mice, iAKO mice also developed severe lipoatrophy 28 days after tamoxifen-induced *Lats1/Lats2* deletion, as measured by decreased body weight (Fig. 1h) and the ratio of fat mass to lean mass (Fig. 1i). Analysis of iWAT from iAKO mice revealed a dramatic reduction in iWAT tissue size (Fig. 1j), accompanied by severe delipidation at this timepoint (Fig. 1k). Both gonadal white adipose tissue (gWAT) and brown adipose tissue (BAT) from both AKO and tamoxifen-treated iAKO mice displayed a similar lipoatrophic phenotype as observed in iWAT (Extended Data Fig. 2). Notably, similar to standard chow diet-fed iAKO mice, tamoxifen-induced deletion of adipose *Lats1/Lats2* in high-fat diet-fed iAKO mice also resulted in a significant reduction in fat mass (Extended Data Fig. 3). We further validated this phenotype using in vitro systems, including 4-hydroxytamoxifen-treated adipocytes differentiated from the SVF of iAKO mice (Fig. 1l–n), primary adipocytes isolated from tamoxifen-treated iAKO mice (Extended Data Fig. 4a,b) and C3H10T1/2 adipocytes expressing a constitutively active form of TAZ (Extended Data Fig. 4c–e). In each case, the cultured cells lost their ability to maintain the mature adipocyte state and acquired a delipidated fibroblast-like morphology with a marked reduction in the expression of adipocyte-specific genes. Collectively, these findings demonstrate the necessity of LATS1/LATS2 kinases for maintaining the mature adipocyte state and that adipocyte-specific activation of YAP/TAZ by *Lats1/Lats2* deletion causes lipoatrophy by regressing mature adipocytes to fibroblast-like cells.

### PPARG agonism reverses lipoatrophy induced by YAP/TAZ

**Fig. 2:**
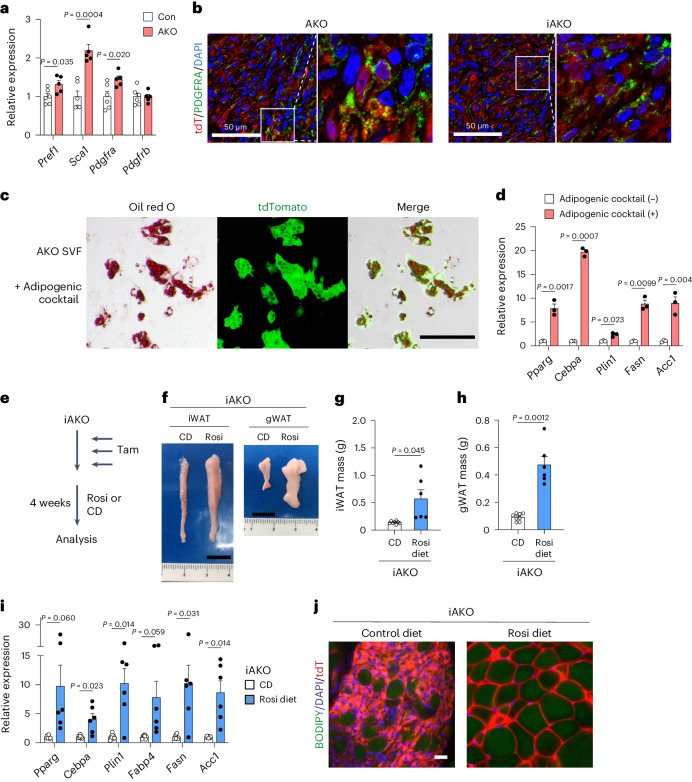
PPARG agonism rescues lipoatrophy in adipose-specific *Lats1/Lats2* knockout mice. **a**, RT–qPCR analysis of adipocyte progenitor marker genes in iWAT of 4-week-old Con and AKO mice (*n* = 6 Con, *n* = 5 AKO). **b**, Immunofluorescence staining of tdT (red) and PDGFRA (green) in iWAT of 4-week-old AKO; tdT mice and 8- to 10-week-old iAKO mice at 1 month after the final tamoxifen injection. Nuclei were stained with DAPI (blue). Boxed region shown at higher magnification on right. **c**, Adipocytes differentiated from the SVF of iWAT of 4-week-old AKO; tdT mice were stained with Oil Red O and subjected to bright-field (Oil Red O) or fluorescence (tdT) microscopy. Scale bar, 50 µm. **d**, RT–qPCR analysis of mature adipocyte marker genes in the SVF from iWAT of AKO mice treated (+), or not treated (−), with an adipogenic cocktail as in **c** (*n* = 3 per group). **e**–**j**, Tamoxifen (Tam)-treated iAKO mice were maintained on a diet containing rosiglitazone (Rosi) or a control diet (CD) for 4 weeks (**e**), after which gross morphology of iWAT and gWAT (scale bar, 1 cm) (**f**), iWAT mass (**g**) and gWAT mass (**h**) were analysed. RT–qPCR analysis of adipocyte marker genes (**i**) and whole-mount fluorescence imaging of tdT (red), BODIPY (green) and Hoechst (blue) of iWAT (scale bar, 20 µm) (**j**). (*n* = 8 CD, *n* = 6 Rosi diet). Source data

**Extended Data Fig. 5:**
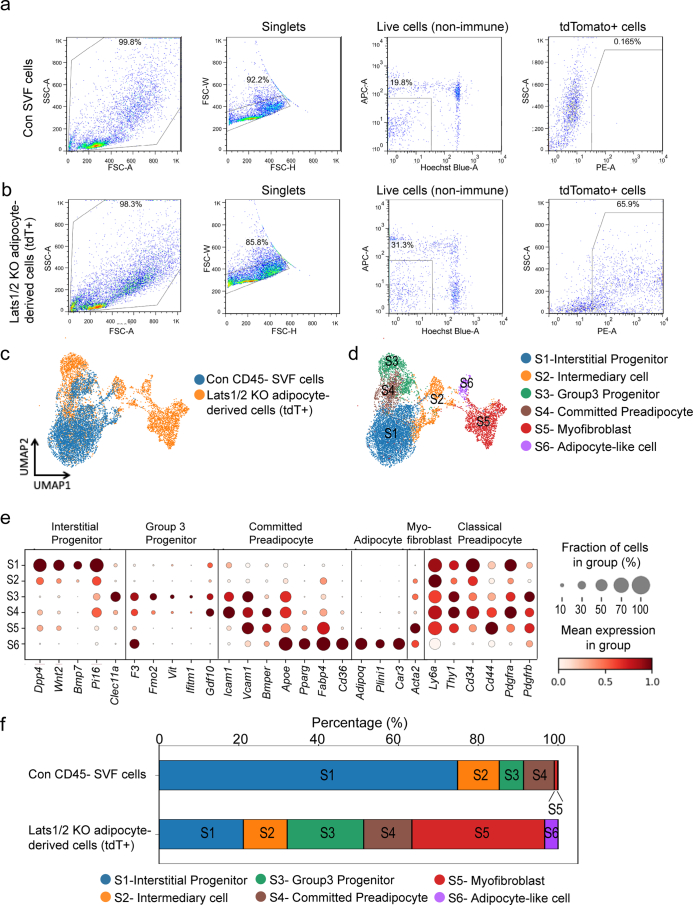
Lats1/2 KO adipocyte-derived cells acquire progenitor-like gene expression signatures. **a-f**, Stromal vascular fraction (SVF) were isolated from the inguinal white adipose tissue of 4-week-old *Lats1*^*fl/fl*^*; Lats2*^*fl/fl*^*; Rosa-LSL-tdTomato* (Con) and *Adipoq-Cre; Lats1*^*fl/fl*^*; Lats2*^*fl/fl*^*; Rosa-LSL-tdTomato* (AKO; tdT) male mice (n = 10 Con, n = 5 AKO; tdT). CD45-/tdT- cells from Con SVF (Con CD45- SVF cells) and CD45-/tdT+ cells from AKO; tdT SVF (Lats1/2 KO adipocyte-derived cells) were flow-sorted and subjected to single cell RNA-sequencing. Doublets were excluded based on forward scatter profiles. Live cells were selected using DAPI (Hoechst Blue) and leukocytes were removed using CD45 (APC) antibody. FACS gating strategy for Con CD45- SVF cells (**a**), and Lats1/2 KO adipocyte-derived cells (**b**). Uniform manifold approximation and projection (UMAP) (**c**), clusters (**d**) of Con CD45- SVF cells and Lats1/2 KO adipocyte-derived cells. Dot plot analysis of expression levels for adipocyte progenitor subtype, adipocyte, and classical preadipocyte marker genes in each cluster (Merrick et al. Science 2019) (**e**). Distributions of Con CD45- SVF cells and Lats1/2 AKO adipocyte derived cells (**f**). Source data

**Extended Data Fig. 6:**
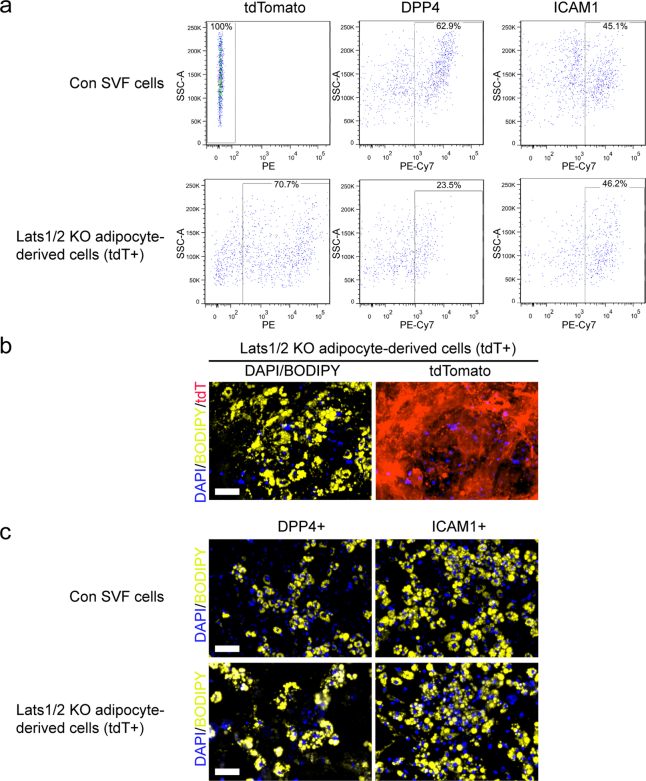
Lats1/2 KO adipocyte-derived cells express cell surface progenitor markers and retain adipogenic potential to redifferentiate into adipocytes. **a-c**, Stromal vascular fraction (SVF) were isolated from the iWAT of 4-week-old *Lats1*^*fl/fl*^*; Lats2*^*fl/fl*^*; Rosa-LSL-tdTomato* (Con) and *Adipoq-Cre; Lats1*^*fl/fl*^*; Lats2*^*fl/fl*^*; Rosa-LSL-tdTomato* male mice (n = 5 Con, n = 3 AKO). CD45-/CD31-/tdT- cells from control SVF (Con SVF cells) and CD45-/CD31-/tdT+ cells from AKO; tdT SVF (Lats1/2 KO adipocyte-derived cells) were selected, followed by flow-sorting with DPP4 or ICAM1 antibody. 48 hr after plating, the cells were subjected to adipogenic differentiation media to test adipogenic potential. FACS gating plot using DPP4 (PE-Cy7) or ICAM1 (PE-Cy7) antibody of Con SVF cells or Lats1/2 KO adipcoyte-derived cells (**a**). Fluorescence images of adipocytes differentiated from Lats1/2 KO adipocyte-derived cells (**b**). Fluorescence images of adipocytes differentiated from DPP4+ or ICAM1+ cells from Con SVF cells or Lats1/2 KO adipocyte-derived cells (**c**). tdT, red; BODIPY, yellow; DAPI, blue. Scale bar = 50 μm. Experiment was repeated twice with similar results. Source data

Hippo–YAP/TAZ signalling plays a key role in tissue regeneration, facilitating stem cell renewal or cell dedifferentiation^20^. Hence, we evaluated whether the iWAT of AKO mice might have acquired progenitor markers. Indeed, quantitative PCR with reverse transcription (RT–qPCR) analysis revealed increased expression of adipocyte progenitor marker genes such as *Dlk1* (*Pref1*), *Ly6a* (*Sca1*) and *Pdfgra* in LATS1/LATS2-deficient iWAT (Fig. 2a). Immunostaining of iWAT sections from AKO and tamoxifen-treated iAKO mice confirmed that tdT^+^ cells expressed platelet-derived growth factor receptor alpha (PDGFRA) and the proliferation marker Ki67 (Fig. 2b and Supplementary Fig. 1). Single-cell RNA-sequencing (RNA-seq) profiles of tdT^+^ cells isolated from the iWAT of AKO mice revealed that the gene expression signatures of tdT^+^ cells overlapped with those of adipocyte progenitor populations^21^ (Extended Data Fig. 5), further supporting the notion that the LATS1/LATS2-deficient adipocyte-derived cells had acquired progenitor-like traits. We next examined whether these cells retained the lineage potential to redifferentiate into lipid-bearing adipocytes. Culturing the SVF of iWAT from AKO mice with an adipogenic cocktail resulted in the differentiation of tdT^+^ *Lats1/Lats2* knockout cells into lipid-bearing adipocytes, as indicated by positive staining with Oil Red O and increased expression of adipogenic marker genes (Fig. 2c,d). Furthermore, we examined the adipogenic potential of specific subpopulations isolated from the SVF of AKO iWAT based on established adipocyte progenitor cell surface markers, DPP4 and ICAM1, which mark interstitial progenitor cells and preadipocyte cells, respectively^21,22^. We found that both DPP4-positive and ICAM1-positive subpopulations sorted from tdT^+^ *Lats1/Lats2* knockout cells could undergo redifferentiation to adipocytes (Extended Data Fig. 6). Given the adipogenic potential of these cells in vitro and the inhibitory function of TAZ on PPARG activity^15,17^, we reasoned that treatment with a PPARG agonist could potentially reverse the lipoatrophic phenotype of tamoxifen-treated iAKO mice. To explore this, we administered a diet containing rosiglitazone to iAKO mice for 4 weeks following tamoxifen-induced *Lats1/Lats2* deletion (Fig. 2e). Analysis of these mice revealed significantly increased adipose tissue size and mass (Fig. 2f–h), and increased expression of adipocyte-specific genes (Fig. 2i) compared to tamoxifen-treated iAKO mice fed a control diet. Epifluorescence images of iWAT from rosiglitazone-treated iAKO mice containing the *Rosa-LSL-tdTomato* Cre reporter revealed that all lipid droplet-bearing adipocytes were tdT^+^ (Fig. 2j). This observation indicates the recovery of adipose tissue mass in iAKO mice treated with rosiglitazone resulted from the process of redifferentiation of mutant cells into mature adipocytes, rather than de novo adipogenesis originating from non-mutant endogenous progenitor cells. Together, these results demonstrate that LATS1/LATS2-deficient adipocytes acquire progenitor-like characteristics while retaining adipogenic potential and the ability to reverse lipoatrophy in response to PPARG agonism.

### Lipoatrophic iAKO mice are spared from metabolic dysfunction

**Fig. 3:**
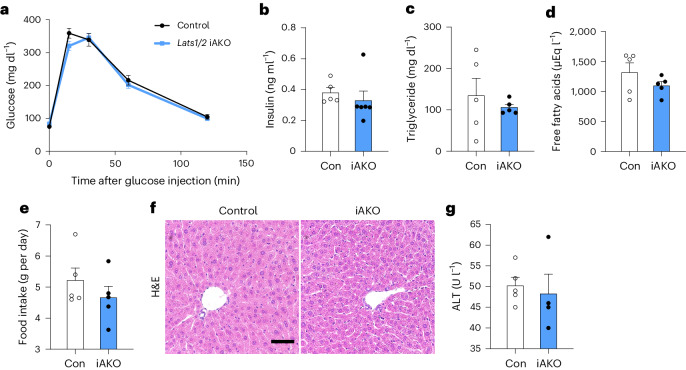
Adipose-specific *Lats1/Lats2* knockout mice do not display lipodystrophy-related metabolic dysfunction despite severe lipoatrophy. Eight- to ten-week-old Con and iAKO mice were analysed at 3–4 weeks after the final tamoxifen injection. **a**,**b**, Glucose tolerance test (**a**) and fasting serum insulin (**b**) (*n* = 5 Con, *n* = 6 iAKO). **c**–**g**, Fasting serum triglyceride (**c**), fasting serum free fatty acids (**d**), food intake (body weight) (**e**), liver histology (scale bar, 50 µm) (**f**) and serum ALT (**g**) (*n* = 5 Con, *n* = 5 iAKO). Source data

**Extended Data Fig. 7:**
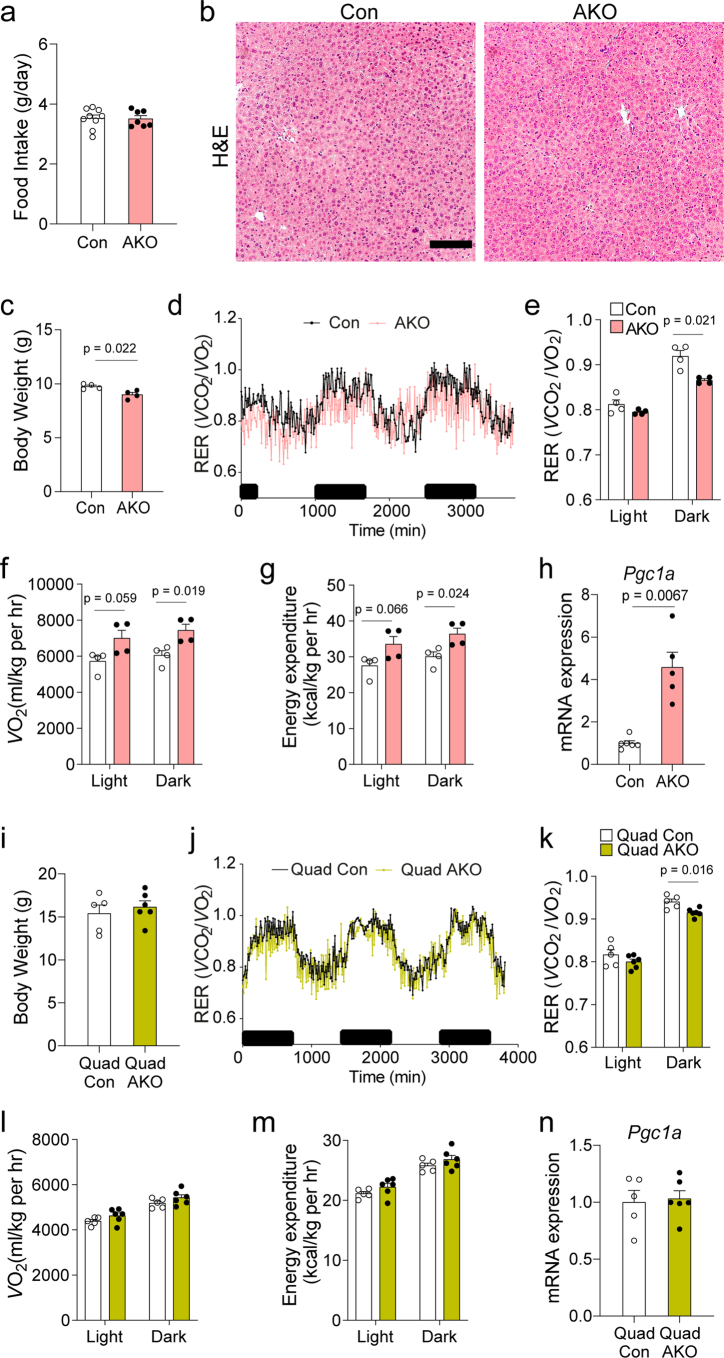
Adipose Yap/Taz is essential for adipose Lats1/2 deletion induced whole-body energy expenditure and hepatic Pgc1a induction. **a-b**, Food intake (**a**) and liver H&E histology (scale bar = 100μm) (**b**) of *Lats1*^*fl/fl*^; *Lats2*^*fl/fl*^ (Con) or *Adipoq-Cre*; *Lats1*^*fl/fl*^; *Lats2*^*fl/fl*^ (AKO) male mice at 4 weeks of age. (n = 9 Con, n = 7 AKO) **c-h**, Con and AKO were placed in a metabolic chamber for indirect calorimetry at 4 weeks of age. Con and AKO mice were sacrificed at 4-5 weeks of age. Body weight (**c**), RER over 3 days (**d**), during the combined light or dark periods (**e**) oxygen consumption rate (VO2) (**f**) and energy expenditure (**g**) (n = 4 per genotype). Liver *Pgc1a* mRNA expression (**h**) was assessed by qPCR (n = 6 Con, n = 5 AKO). **i-n**, *Lats1*^*fl/fl*^*; Lats2*^*fl/fl*^*; Yap*^*fl/fl*^*; Taz*^*fl/fl*^ (Quad Con) and *Adipoq-Cre; Lats1*^*fl/fl*^*; Lats2*^*fl/fl*^*; Yap*^*fl/fl*^*; Taz*^*fl/fl*^ (Quad AKO) male mice were placed in a metabolic chamber for indirect calorimetry at 4 weeks of age. Quad Con and Quad AKO male mice were sacrificed at 4-5 weeks of age. Body weight (**i**), RER over 3 days (**j**), during the combined light or dark periods (**k**) oxygen consumption rate (VO2) (**l**) and energy expenditure (**m**). Liver Pgc1a mRNA expression (**n**) was assessed by qPCR (n = 5 Quad Con, n = 6 Quad AKO). Data expressed as mean ± s.e.m. Data were analyzed by two-tailed unpaired t test. Source data

Lipoatrophy is commonly associated with adverse metabolic outcomes, partly due to insufficient production of adipokines^5^. Moreover, reduced energy storage capacity associated with lipoatrophy results in lipid spillover into plasma and to the liver, which can cause impaired glucose homeostasis^1,4,5^. Given that adipocyte-specific *Lats1/Lats2* knockout mice displayed a severe loss of adipose tissue, we next evaluated various metabolic parameters in these animals. Unexpectedly, tamoxifen-treated iAKO mice did not show altered glucose tolerance (Fig. 3a). In contrast to previously described mouse models of lipodystrophy^5,23,24^, the iAKO mice showed no changes in fasting serum concentrations of insulin, triglycerides, free fatty acids and food intake (Fig. 3b–e). Moreover, neither liver steatosis nor hepatotoxicity was apparent in iAKO mice by histological analysis or measurement of serum alanine aminotransferase (ALT), respectively (Fig. 3f,g). Similarly, AKO mice also did not show increased food intake or liver steatosis phenotypes despite being severely lipoatrophic (Extended Data Fig. 7a,b).

**Fig. 4:**
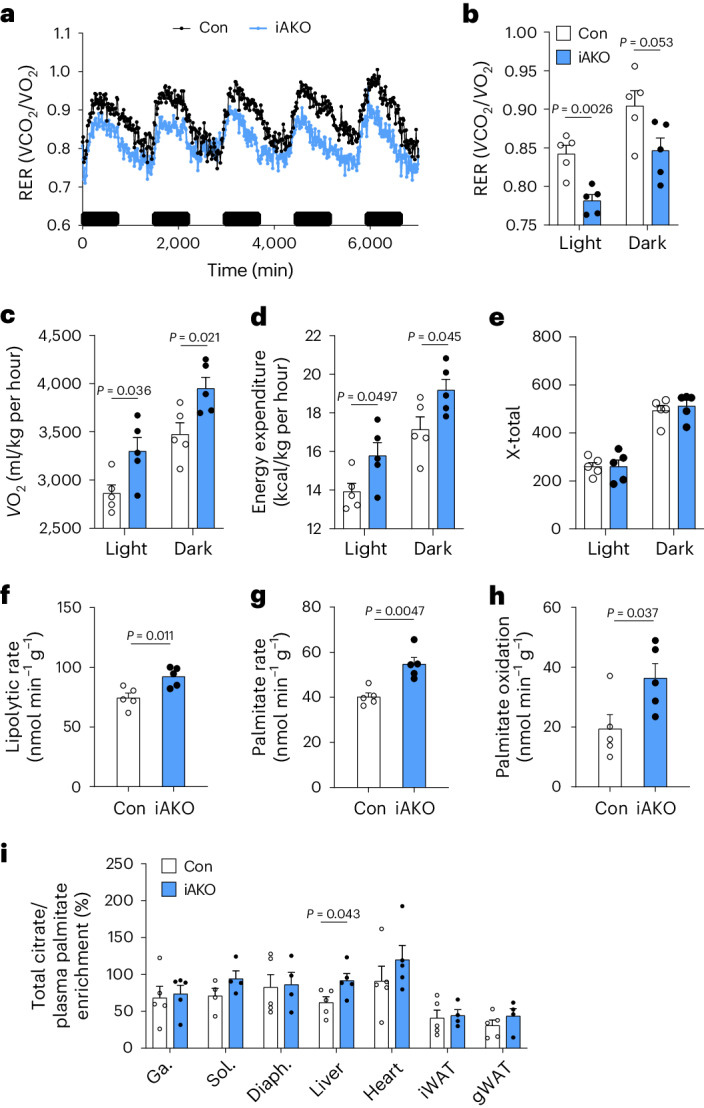
Adipose-specific *Lats1/Lats2* knockout mice have increased energy expenditure and fat oxidation. **a**–**e**, Eight- to ten-week-old Con and iAKO mice were analysed in metabolic chambers for 5 days, starting 1 day after the final tamoxifen treatment. RER over 5 days (**a**) and during the combined light or dark periods (**b**), oxygen consumption rate (*V*O_2_) (**c**), energy expenditure (**d**) and total horizontal motor activity (X-total) (**e**). **f**–**h**, Eight- to ten-week-old Con and iAKO mice were analysed for metabolic flux at 1 day after the final tamoxifen injection. Rate of lipolysis (glycerol release rate) (**f**), rate of palmitate turnover (**g**), palmitate oxidation rate (**h**) and ^13^C-labelled palmitate-derived citrate enrichment in various tissues (**i**). Ga., gastrocnemius; Sol., soleus; Diaph., diaphragm. (*n* = 5 Con, *n* = 5 iAKO). Source data

To characterize the effects of adipose Lats1/Lats2 deletion on whole-body energy homeostasis, we performed metabolic chamber analysis starting 1 day after the final tamoxifen injection, when there was no difference in body weight between control and iAKO mice (Supplementary Fig. 2). Metabolic chamber analysis revealed that tamoxifen-treated iAKO mice had a lower respiratory exchange ratio (RER; Fig. 4a,b) in addition to increased oxygen consumption (Fig. 4c) and energy expenditure (Fig. 4d) compared to control mice. This suggested that iAKO mice had increased energy expenditure and utilization of fatty acids as their preferred energy source, which could explain the lack of excess lipid in the liver and serum despite severe lipoatrophy. Other energy-consuming factors, such as locomotor activity, showed no significant difference between control and iAKO mice (Fig. 4e). In addition, the expression of brown fat-specific genes including *Ucp1* and *Cidea* was reduced in the BAT of iAKO mice (Extended Data Fig. 2l,n), suggesting that BAT thermogenesis did not contribute to the increased energy expenditure of iAKO mice. We next performed in vivo metabolite tracing with ^13^C-labelled palmitate and ^2^H-labelled glycerol to measure the metabolic flux of lipid nutrients. Consistent with the reduced RER of iAKO mice, stable isotope tracing revealed an increased rate of lipolysis (Fig. 4f), palmitate turnover (Fig. 4g) and subsequent palmitate oxidation (Fig. 4h) in these mice. Notably, ^13^C-labelled citrate was significantly enriched in the liver but not in muscle and fat tissues of iAKO mice (Fig. 4i), indicating that adipocyte-specific *Lats1/Lats2* deletion enhances fatty acid oxidation (FAO) in the liver compared to other metabolically active tissues. These findings suggest that increased energy expenditure and FAO, particularly in the liver, protects iAKO mice from developing lipodystrophy-associated metabolic dysfunction, such as fatty liver and glucose intolerance. We also observed an increase in oxygen consumption, energy expenditure and hepatic *Ppargc1a* (*Pgc1a*) expression, a marker for FAO, in AKO mice (Extended Data Fig. 7c–h). However, these changes were not observed in Quad AKO mice (Extended Data Fig. 7i–n), indicating that adipose YAP/TAZ functions downstream of LATS1/LATS2 in regulating not only adipose plasticity (Extended Data Fig. 1f) but also whole-body energy expenditure (Extended Data Fig. 7i–n).

### YAP/TAZ activation uncouples leptin levels from fat mass

**Fig. 5:**
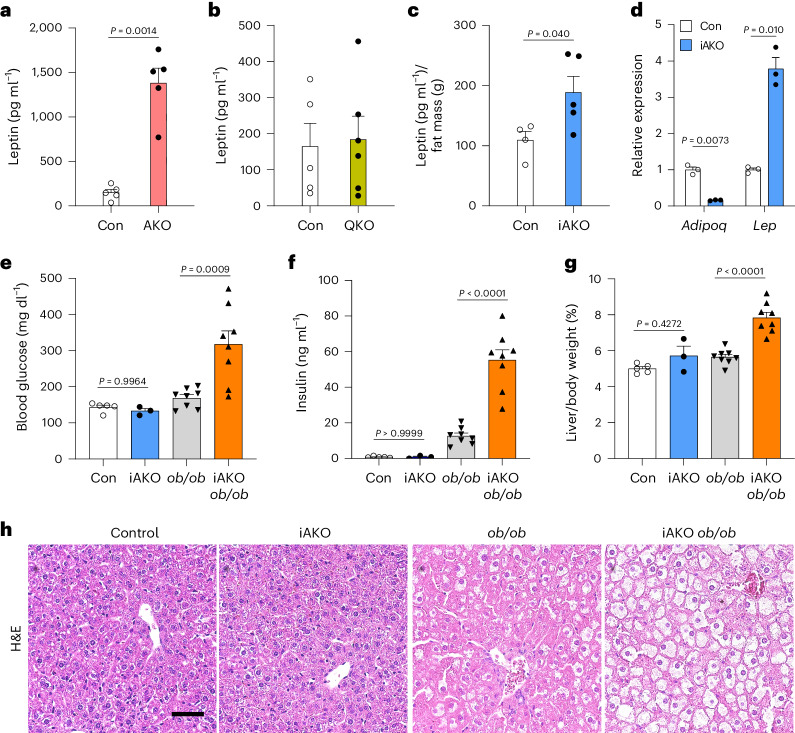
Leptin upregulation is essential for the prevention of lipodystrophy-associated metabolic dysfunction in adipose-specific *Lats1/Lats2* knockout mice. **a**, Serum leptin levels in Con and AKO mice (*n* = 5 Con, *n* = 5 AKO). **b**, Serum leptin levels in Con and Quad AKO (QKO) mice (*n* = 5 Con, *n* = 6 QKO). **c**, Serum leptin levels normalized by fat mass in Con and iAKO mice (*n* = 4 Con, *n* = 5 iAKO). **d**, RT–qPCR analysis of *Adipoq* and *Lep* expression in adipocytes differentiated from the SVF of iWAT from Con or iAKO mice after 4-hydroxytamoxifen treatment in vitro as in Fig. 1l–n (*n* = 3 Con, *n* = 3 iAKO). **e**–**h**, Six-week-old Con, iAKO, *Lep*^ob/ob^ (*ob/ob*) and iAKO *ob/ob* (*Adipoq-CreER*^*T2*^; *Lats1*^fl/fl^; *Lats2*^fl/fl^; *Lep*^ob/ob^) mice at 2 weeks after the final tamoxifen treatment were analysed for fasting blood glucose levels (**e**), fasting serum insulin levels (**f**), liver/body weight ratios (**g**) and liver histology (scale bar, 50 µm) (**h**). (*n* = 5 Con, *n* = 3 iAKO, *n* = 8 *ob/ob*, *n* = 8 iAKO *ob/ob*). Source data

We next sought to identify the factors responsible for the systemic increase in energy expenditure and prevention of lipotoxicity in mice lacking *Lats1/Lats2* in adipose tissue. Adipocytes regulate systemic metabolism in an endocrine manner by secreting adipokines. Among these, circulating leptin levels have been found to be associated with fat mass^8,9^ and play a key role in control of food intake and energy expenditure^25–27^. Moreover, leptin replacement therapy effectively corrects systemic metabolic dysfunction associated with lipodystrophy in mice and humans^6,7,28^. Unexpectedly, despite the near-complete loss of adipose tissue, we found that serum leptin concentrations in AKO mice were ~15-fold higher compared to control (Fig. 5a), whereas this increase was not observed in Quad AKO mice (Fig. 5b). Similarly, serum leptin levels normalized by fat mass were increased ~2-fold in tamoxifen-treated iAKO mice compared to control (Fig. 5c). These results are in striking contrast with other mouse models of lipoatrophy, where serum leptin levels are almost undetectable^5,23,24^. We also observed that inducible *Lats1/Lats2* deletion in mature adipocytes resulted in the upregulation of *Lep* expression, whereas the expression of *Adipoq*, another adipokine, was markedly reduced (Fig. 5d).

### Leptin protects against metabolic dysfunction in iAKO mice

**Extended Data Fig. 8:**
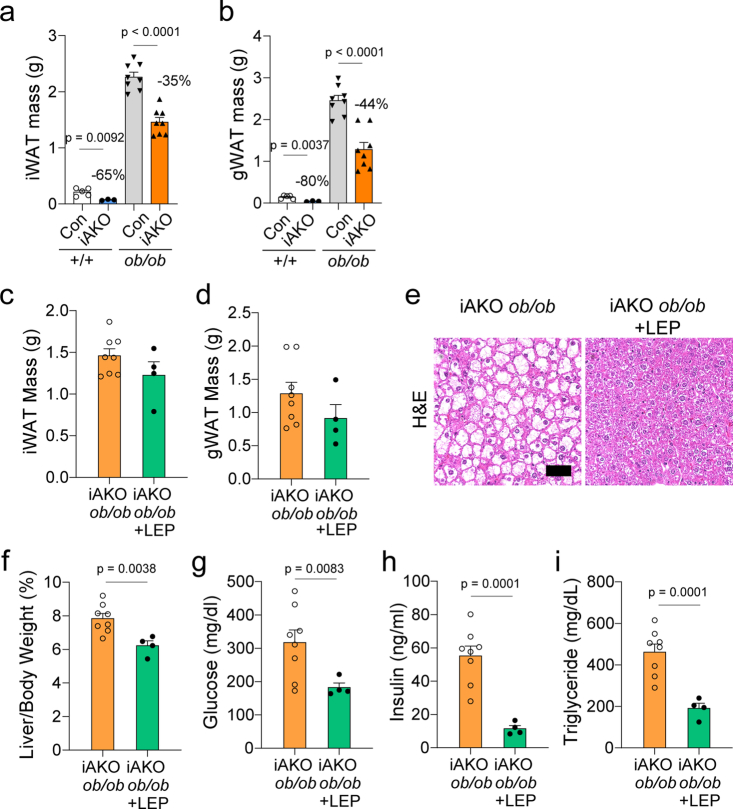
Recombinant leptin replacement rescues lipodystrophy-associated metabolic dysfunction in leptin-deficient adipose-specific Lats1/2 knockout mice. **a-b**, *Lats1*^*fl/fl*^; *Lats2*^*fl/fl*^; *Lep* + */+* (Con), *Adipoq-CreER*; *Lats1*^*fl/fl*^; *Lats2*^*fl/fl*^; *Lep* + */+* (iAKO), *Lats1*^*fl/fl*^; *Lats2*^*fl/fl*^; *Lep*^*ob/ob*^ (Con *ob/ob*), and *Adipoq-CreER*; *Lats1*^*fl/fl*^; *Lats2*^*fl/fl*^; *Lep*^*ob/ob*^ (iAKO *ob/ob*) male and female mice at 5 weeks of age were intraperitoneally injected with tamoxifen (100 mg/kg) every other day for three times. Two weeks after the final tamoxifen injection, mice were sacrificed, followed by analysis of iWAT (**a**) and gWAT (**b**) mass. Data expressed as mean ± s.e.m. (n = 5 Con, n = 3 iAKO, n = 8 *ob/ob*, n = 8 iAKO *ob/ob)* **c-i**, iAKO *ob/ob* male and female mice at 5 weeks of age were intraperitoneally treated with tamoxifen (100 mg/kg) every other day for three times. From one day after the last tamoxifen injection, iAKO *ob/ob* mice were intraperitoneally injected with 5 mg/kg recombinant leptin (LEP) every day until mice were sacrificed. Two weeks after the final tamoxifen injection, mice were sacrificed, followed by analysis of iWAT mass (**c**), gWAT mass (**d**), liver H&E histology (scale bar = 50 μm) (**e**), liver mass/body mass (**f**), fasting blood glucose (**g**), fasting serum insulin concentration (**h**), and fasting serum triglycerides (**i**), were determined. Data expressed as mean ± s.e.m. (n = 8 iAKO *ob/ob*, n = 8 iAKO *ob/ob* + LEP) Data were analyzed by two-tailed unpaired t test. Source data

Given the potent therapeutic effects of leptin replacement on glucose homeostasis and liver steatosis in lipodystrophic mice and humans^6,7,28^, we speculated whether elevated leptin levels might explain the apparent absence of lipodystrophy-associated metabolic dysfunction in iAKO mice. To examine this possibility, we generated inducible adipocyte-specific *Lats1/Lats2* knockout mice that also lack a functional *Lep* gene (iAKO *ob/ob* mice) and analysed the metabolic phenotypes of these mice following tamoxifen-induced deletion of *Lats1/Lats2*. In this leptin-deficient context, adipocyte LATS1/LATS2 deficiency was still effective in reducing adipose tissue mass (Extended Data Fig. 8a,b). In contrast, tamoxifen-treated iAKO *ob/ob* mice developed severe hyperglycaemia with fasting blood glucose levels reaching ~300 mg dl^−1^, which is ~2-fold higher compared to either iAKO or *ob/ob* mice (Fig. 5e). Strikingly, fasting serum insulin concentrations of iAKO *ob/ob* mice reached ~60 ng ml^−1^, which is ~70-fold and ~15-fold higher compared to iAKO and *ob/ob* mice, respectively (Fig. 5f). Furthermore, examination of liver tissue from iAKO *ob/ob* mice revealed pronounced hepatomegaly and liver steatosis, compared to both *ob/ob* or iAKO mice (Fig. 5g,h). To confirm that leptin deficiency was the key contributor to the metabolic dysfunction in iAKO *ob/ob* mice, we administered recombinant leptin to iAKO *ob/ob* mice. Analysis of these mice showed that recombinant leptin treatment was sufficient to rescue the impaired glucose homeostasis, hyperinsulinaemia and liver steatosis observed in iAKO ob/ob mice (Extended Data Fig. 8c–i), while it did not prevent fat mass reduction by adipose Lats1/Lats2 deletion (Extended Data Fig. 8a,b). These results demonstrate the critical role of leptin in protecting lipoatrophic iAKO mice from metabolic dysfunction.

### YAP/TAZ directly regulate *Lep* transcription

**Fig. 6:**
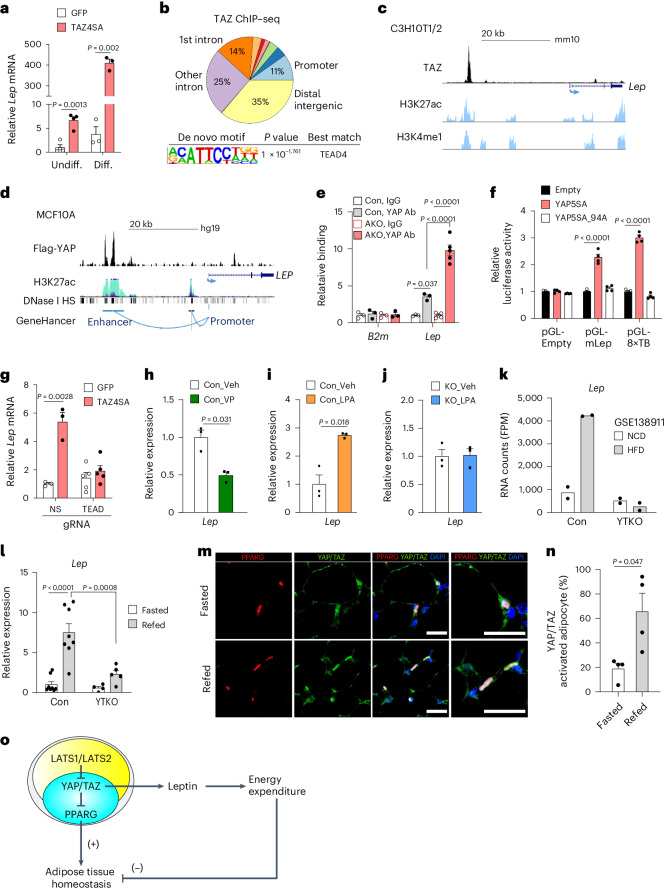
YAP/TAZ–TEAD axis regulates *Lep* expression via directly binding the *Lep* gene enhancer. **a**, *Lep* mRNA expression in undifferentiated (Undiff.) and adipocyte-differentiated (Diff.) C3H10T1/2 cells 2 days after transduction with GFP or TAZ4SA adenovirus (Undiff. *n* = 4 per group, Diff. *n* = 3 per group). **b**,**c**, TAZ ChIP–seq analysis of adipocyte-differentiated C3H10T1/2 cells expressing TAZ4SA. Peak annotation and motif enrichment analysis (**b**), and TAZ binding to *Lep* enhancer. H3K27ac and H3K4me1 data (GSE74189)^33^ (**c**). **d**, Flag-tagged YAP ChIP–seq analysis of MCF10A cells (GSE97972)^34^. YAP binding to *LEP* enhancer. H3K27ac, DNase I hypersensitivity (HS) (ENCODE project) and GeneHancer data^65^. **e**, YAP binding to *Lep* enhancer in SVF-differentiated adipocytes from 4-week-old Con or AKO mice (*B2m* *n* = 3 per group; *Lep* *n* = 3 Con IgG, *n* = 3 Con YAP Ab, *n* = 5 AKO IgG, *n* = 5 AKO YAP Ab) Ab, antibody. **f**, Luciferase reporter assay for *Lep* enhancer (pGL-mLep) or TEAD-binding sequences (pGL-8×TB) (*n* = 4 per group). **g**, *Lep* mRNA expression in C3H10T1/2 cells with CRISPR–Cas9 deletion of *Lep* enhancer TEAD-binding site (TEAD) or non-specific (NS) guide RNA (gRNA) control (NS, *n* = 3 per group, TEAD *n* = 5 per group). **h**–**j**, *Lep* mRNA expression in adipocytes differentiated from C3H10T1/2 cells (Con) or C3H10T1/2 cells with *Lep* enhancer TEAD-binding site deletion (KO). Con treated with vehicle (Veh) or verteporfin (VP) (**h**), Con (**i**) and KO (**j**) treated with Veh or LPA (*n* = 3 per group). **k**, RNA-seq analysis (GSE138911) for *Lep* expression in gWAT of *Yap*^fl/fl^; *Taz*^fl/fl^ (Con) or *Adipoq-Cre; Yap*^fl/fl^; *Taz*^fl/fl^ (YTKO) mice fed normal chow (NCD) or high-fat (HFD) diet. FPM, fragments per million mapped fragments (*n* = 2 per genotype). **l**, *Lep* mRNA expression in gWAT of Con and YTKO mice after overnight food deprivation (Fasted) or 4 h refeeding (Refed) (Con *n* = 8 per group, *n* = 4 YTKO Fasted, *n* = 5 YTKO Refed). **m**,**n**, Immunofluorescence images of YAP/TAZ (green), PPARG (adipocyte nuclei, red) and DAPI (blue) (scale bar, 20 µm) (**m**), and proportion YAP/TAZ-activated adipocytes quantified by nuclear/cytoplasmic ratio of YAP/TAZ per PPARG^+^ nuclei (**n**) in iWAT of C57BL6/J mice (*n* = 4 per group). **o**, Working model of Hippo–YAP/TAZ function in mature adipocytes. Source data

Despite the long-established importance of leptin in adipocyte biology, the molecular mechanisms governing *Lep* gene expression remain unclear. The pronounced increase in serum leptin levels observed in lipoatrophic AKO mice (Fig. 5a), coupled with the observation of normal levels of leptin in Quad AKO mice (Fig. 5b), raised the intriguing possibility of YAP/TAZ involvement in the regulation of leptin gene expression. To explore this, we tested the effect of a constitutively active form of TAZ (TAZ4SA) on *Lep* expression. We observed that adenoviral expression of TAZ4SA induced a marked increase in *Lep* mRNA expression in C3H10T1/2 cells both before and after their differentiation into adipocytes as measured by RT–qPCR (Fig. 6a). YAP/TAZ exert coregulator function by interacting with transcription factors at target gene enhancers, many of which contain consensus TEAD-binding sequences^29^. To examine whether TAZ can bind to the *Lep* locus, C3H10T1/2 cells expressing tamoxifen-inducible TAZ4SA were differentiated into adipocytes and subjected to TAZ ChIP–seq. Annotation of the TAZ ChIP peaks showed that TAZ bound predominantly to intergenic regions and introns rather than to promoters and, consistent with previous YAP/TAZ studies using chromatin immunoprecipitation followed by sequencing (ChIP–seq)^30,31^, that TEAD4 was the most significantly enriched motif (Fig. 6b). Importantly, we found that TAZ bound to a region located 28 kb upstream of the transcription start site of mouse *Lep*, which contains a conserved TEAD-binding element (Fig. 6c). This TAZ-binding region was found to colocalize with the active enhancer site of *Lep* identified in mouse adipocytes as marked by histone H3 monomethylated at Lys4 (H3K4me1) and histone H3 acetylated at Lys27 (H3K27ac)^32,33^.

To examine YAP/TAZ binding to the leptin gene enhancer in another system, we analysed ChIP–seq data for Flag-tagged YAP in the human mammary gland epithelial cell line MCF10A (ref. ^31^). The MCF10A ChIP–seq profile revealed that YAP bound to a conserved region located 24 kb upstream of the human *LEP* gene (Fig. 6d). Of note, the YAP-binding region was also associated with the open chromatin mark H3K27ac and a DNase I hypersensitivity cluster. This region has previously been implicated as an enhancer that interacts with the *LEP* promoter^34^.

While the role of TAZ has been emphasized more than that of YAP in adipocytes, our results indicated that both YAP and TAZ bind to a conserved enhancer region of the leptin gene. Moreover, our YAP ChIP–qPCR analysis showed that the enhancer region of the *Lep* gene, but not the non-specific *B2m* control gene, was specifically enriched with YAP in SVF-differentiated adipocytes from iWAT of 4-week-old control or AKO mice (Fig. 6e). We noted that the YAP/TAZ-binding region overlapped with the 5′ region of the genomic locus encoding the long noncoding RNA (lncRNA) *LncOb* (also known as *Lnc-leptin*), which has been shown to regulate leptin mRNA expression^35,36^. To investigate whether YAP/TAZ regulate *LncOb* expression in adipocytes, we examined *LncOb* expression in cells differentiated from the SVF of AKO mice and adipocytes differentiated from C3H10T1/2 cells transduced with TAZ4SA-expressing adenovirus. While both *LncOb* RNA and *Lep* mRNA levels were increased after differentiation of SVF or C3H10T1/2 cells into adipocytes (Supplementary Fig. 3a), the expression of *LncOb* RNA was not affected by activating YAP/TAZ in either cell system (Supplementary Fig. 3b). These results suggest that YAP/TAZ induce *Lep* mRNA transcription through mechanisms independent of *LncOb* expression.

We next investigated whether YAP/TAZ binding to the *Lep* enhancer results in gene transactivation. A mouse genomic DNA fragment containing the TAZ-binding region of the *Lep* enhancer was cloned into a luciferase reporter construct (pGL-mLep) and transfected into 293T cells. Forced expression of YAP5SA, a constitutively active form of YAP, markedly induced luciferase activity of pGL-mLep as well as that of a positive control reporter plasmid containing eight tandem TEAD-binding sites (pGL-8×TB; Fig. 6f). In contrast, YAP5SA_94A, a YAP mutant deficient in TEAD binding, did not activate either of the reporter constructs, suggesting that the TEAD-binding activity of YAP is required for transactivation of the *Lep* enhancer.

To investigate the *cis*-element requirements of the putative YAP/TAZ–TEAD axis that regulate *Lep* transcription, we deleted the TEAD-binding sequence within the *Lep* enhancer via CRISPR–Cas9 genome editing in C3H10T1/2 cells (Supplementary Fig. 4). We found that TAZ4SA failed to induce *Lep* expression in these cells (Fig. 6g), indicating that this TEAD-binding sequence is essential for the upregulation of *Lep* expression by TAZ. Furthermore, we found that verteporfin, a YAP/TAZ inhibitor^37^, reduces *Lep* expression (Fig. 6h) while lysophosphatidic acid, a YAP/TAZ activator^38^, increases *Lep* expression (Fig. 6i) and that this regulation requires the presence of the intact TEAD-binding sequence within the *Lep* enhancer (Fig. 6j). Together, these data show that YAP and TAZ directly regulate *Lep* transcription through a TEAD-binding site on the active enhancer region of the *Lep* gene.

**Extended Data Fig. 9:**
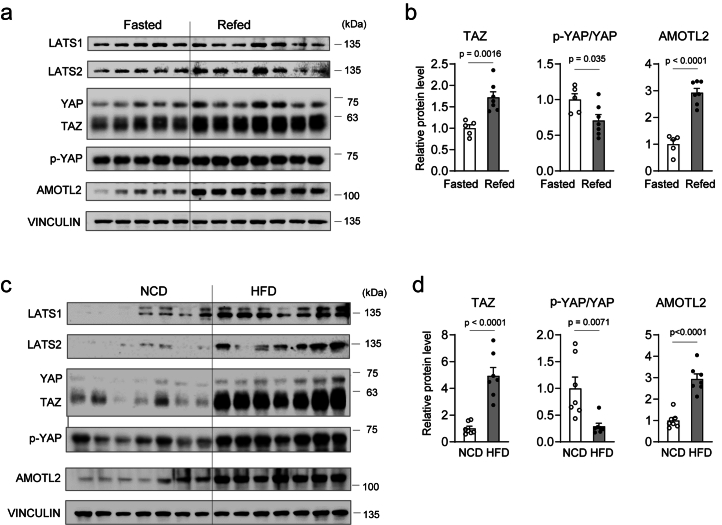
YAP and TAZ in mice adipose tissues are stabilized by a refeed after overnight fasting or high fat diet feeding. **a-b**, Wild type control male mice aged 8 to 10 weeks were fasted overnight and then fed for 4 hours. After fasting or refeeding, inguinal white adipose tissue of mice was collected and subjected to western blot analysis to measure protein levels (**a**). The western signals were quantified against VINCULIN (**b**) (n = 5 Fasted, n = 7 Refed). **c-d**, Wild type control male mice aged at 8 weeks of age were fed with normal chow diet (NCD) or high fat diet (HFD) for 14 weeks. Inguinal white adipose tissue of mice was harvested, and the protein levels were measured by western blot analyses (**c**). The western blot signals were quantified against VINCULIN (**d**) (n = 7 NCD, n = 7 HFD). Data expressed as mean ± s.e.m. Data were analyzed by two-tailed unpaired t test. Source data

The identification of YAP/TAZ as direct regulators of *Lep* gene expression prompted us to investigate their role in regulating endogenous *Lep* expression in various physiological contexts. Analysis of previously published RNA-seq data^18^ revealed that a high-fat diet induces *Lep* expression in mouse adipose tissue in a YAP/TAZ-dependent manner (Fig. 6k). Additionally, we observed that the upregulation of *Lep* expression in mouse adipose tissue following refeeding was also blunted in adipose-specific YAP/TAZ knockout mice (Fig. 6l), indicating the necessity of YAP/TAZ for post-prandial leptin induction. Consistent with a role for YAP/TAZ in *Lep* gene regulation, we observed that *Lep* mRNA expression and the proportion of adipocytes exhibiting nuclear-localized YAP/TAZ, a marker of activated YAP/TAZ, were increased in adipose tissue of wild-type mice following refeeding (Fig. 6m,n). We next conducted western blot analysis of Hippo–YAP/TAZ signalling components in the adipose tissue of wild-type mice subjected to refeeding after overnight fasting or high-fat diet feeding conditions. We observed a significant decrease in phospho-YAP/YAP ratios and a marked increase in the levels of YAP/TAZ and *AMOTL2*, a YAP/TAZ target gene, under both conditions (Extended Data Fig. 9). These results demonstrate that adipose tissue responds to positive energy balance by activating YAP/TAZ, concomitant with reduced LATS1/LATS2 activity.

**Extended Data Fig. 10:**
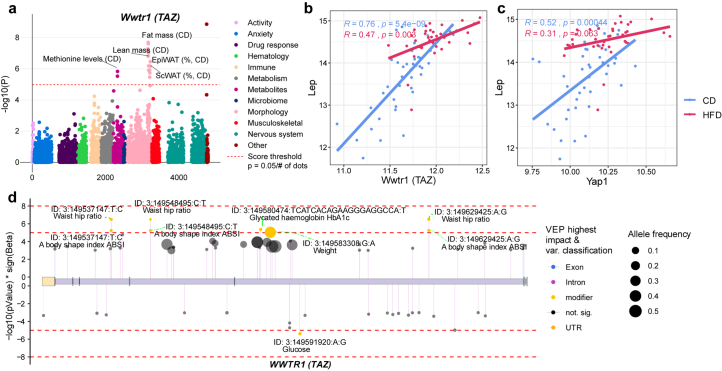
Systems genetics analysis of TAZ expression and genetic variants. **a**, Expression-based Phenome-Wide Association study (ePheWAS) (https://systems-genetics.org) was performed in BXD mice showing the association of *Wwrt1*/TAZ expression in subcutanenous white adipose tissue (ScWAT) and various mouse phenotypes39. The dashed horizontal line represents the significance threshold. CD: chow diet. **b-c**, Scatter plots of *Lep* and *Wwtr1*/TAZ (**b**) or *Yap1*/YAP (**c**) expression in BXD ScWAT under chow diet (CD) and high-fat diet (HFD) conditions. The lines indicate linear fits, and Pearson correlation coefficients are shown. **d**, Lollipop plot demonstrating the associated phenotypes of genetic variants in TAZ identified by genome-wide association study (GWAS) using UK Biobank whole-genome seqence (WGS) data. The variant effect prediction (VEP) is shown. Source data

To further explore the physiological relevance of adipose YAP/TAZ and its role in metabolic regulation, a systems genetics analysis of YAP/TAZ expression and genetic variants in mice and humans was performed. Expression-based phenome-wide association study (ePheWAS) analysis using BXD recombinant inbred mouse panels^39^ revealed a significant association of TAZ expression in subcutaneous WAT with both fat mass and lean mass phenotypes (Extended Data Fig. 10a). Consistent with a role for YAP/TAZ upregulating leptin expression, BXD mouse subcutaneous WAT transcriptome data revealed a positive correlation between YAP/TAZ and leptin mRNA expression (Extended Data Fig. 10b,c). Genome-wide association study (GWAS) analysis using the UK Biobank whole-genome sequencing (WGS) data revealed significant associations between human *TAZ* genetic variants and adipose tissue-related parameters, such as body weight, waist–hip ratio (WHR) and body shape index, as well as HbA1c levels, a key indicator of long-term glycaemic control (Extended Data Fig. 10d). Collectively, these data demonstrate that YAP/TAZ function as physiological regulators of *Lep* expression in response to changes in systemic energy status.

## Discussion

Adipose tissue is a key organ in the regulation of whole-body energy balance^1^. Leptin is produced and secreted from fat tissue upon positive energy balance to maintain organismal energy homeostasis^40^. However, the molecular mechanism linking leptin expression to adipose tissue mass has remained poorly understood. In this study, we investigated mice with adipocyte-specific deletion of *Lats1/Lats2* to reveal that Hippo–YAP/TAZ signalling in mature adipocytes functions on two distinct axes: a YAP/TAZ–TEAD axis that increases systemic energy expenditure via upregulation of leptin expression and a YAP/TAZ-PPARG axis that reduces adipose tissue mass via PPARG target gene repression (Fig. 6o).

Both AKO and iAKO mice develop lipoatrophy characterized by a severe reduction in adipose tissue mass. Sustained activation of YAP/TAZ in mature adipocytes caused them to regress, first into smaller adipocytes and eventually into progenitor-like cells. TAZ blocks adipogenesis by inhibiting PPARG^15^, and adipose-specific TAZ ablation increases PPARG activity^17^, suggesting that TAZ is a key regulator of PPARG activity in adipocytes. We show that PPARG agonism effectively reversed lipoatrophy in iAKO mice, providing in vivo evidence that the balance between YAP/TAZ and PPARG activity regulates adipocyte plasticity and adipose tissue mass.

Lipodystrophy, characterized by a deficiency in fat tissue, is associated with adverse metabolic changes such as fatty liver, hypertriglyceridaemia and insulin resistance^4,5^. Surprisingly, despite being fatless, AKO and iAKO mice do not exhibit metabolic dysfunction associated with lipodystrophy. Interestingly, these lipoatrophic mice have elevated serum leptin levels, which likely contribute to their apparently normal glucose homeostasis and absence of ectopic lipid accumulation. Our finding that iAKO *ob/ob* mice show impaired glucose homeostasis and pronounced liver steatosis strongly supports this interpretation.

Adipocyte YAP/TAZ become activated in response to a high-fat diet^18^, and adipocyte-specific TAZ knockout mice display reduced circulating leptin and *Lep* mRNA levels compared to control mice^17,41^. In this study, we further demonstrate that adipocyte YAP/TAZ are also activated during fasting and refeeding, suggesting their potential role in mediating leptin induction in response to positive energy balance. However, the precise mechanisms by which adipocyte YAP/TAZ activity is regulated by energy balance remain to be fully elucidated. Leptin expression is known to be upregulated by feeding-related signals such as insulin or glucose^40,42^, which have also been implicated as upstream activation signals for YAP/TAZ in other cell types^43,44^. Insulin or insulin-mediated glucose uptake provides a lipogenic signal for the storage of excess energy as triglycerides in adipocytes^45^, and thus it is conceivable that mechanical tension induced by lipid droplet expansion within adipocytes may influence Hippo–YAP/TAZ signalling. These interconnected pathways suggest a complex network through which adipocyte YAP/TAZ may integrate signals related to energy balance to regulate leptin expression and adipose tissue function.

The adipostat hypothesis posits that body fat mass is under homeostatic control of a multi-organ network that balances energy intake and energy expenditure^46,47^. Our discovery that YAP/TAZ coordinates the regulation of adipose tissue mass with systemic energy balance suggests that adipocyte YAP/TAZ function as a previously unrecognized peripheral component of the adipostat. These findings provide a rationale for developing therapeutic interventions that target this pathway to achieve homeostatic fat mass reduction. In conclusion, our study sheds light on the potent control that adipocyte YAP/TAZ exerts over the regulation of fat mass, acting through two distinct transcriptional axes that work in concert to regulate adipose energy storage and systemic energy expenditure.

## Methods

### Mice

*Lats1*^fl/fl^ mice (024941, The Jackson Laboratory), *Lats2*^fl/fl^ mice^48^ and *Rosa26-LSL-tdTomato* mice (007914, The Jackson Laboratory), *Lep*^ob/+^ mice (000632, The Jackson Laboratory), *Yap*^*fl/fl*^ mice^49^, *Taz*^*fl/fl*^ mice^50^, *Adipoq-Cre* (010803, The Jackson Laboratory) and *Adipoq-CreER*^*T2*^ transgenic mice (024671, The Jackson Laboratory) were bred to generate mice used in this study. Tamoxifen (13258, Cayman Chemical) was dissolved in corn oil (C8267, Sigma) and administered via intraperitoneal injection to mice every other day for three doses of 100 mg per kg body weight tamoxifen to induce CreER^T2^ activity. Littermates with control genotypes served as the control group. Male mice were used in all studies except for experiments in Figs. 2c,d and 6e, and Fig. 5, which included both male and female mice. All mice were housed in a specific pathogen-free facility within the Korea Advanced Institute of Science and Technology Laboratory Animal Resource Center. Mice were maintained under a 12-h light–dark cycle and given free access to chow diet (2018, Teklad), chow diet containing 5 mg per kg body weight rosiglitazone (122320-73-4, Adooq Bioscience) or diet containing 60 kcal% fat (D12492, Research Diets) and water. All protocols for mouse experiments were approved by the Institutional Animal Care and Use Committee of the Korea Advanced Institute of Science and Technology.

### Isolation of SVF and primary adipocytes

Adipose tissues were dissected, finely chopped with a razor blade, and digested in Krebs–Ringer–Henseleit buffer (30 mM HEPES acid at pH 7.4, 1 mM CaCl_2_, 120 mM NaCl, 4 mM KH_2_PO_4_, 1 mM MgSO_4_, 10 mM Na_2_CO_3_, 200 nM adenosine and glucose at 0.9 mg ml^−1^) supplemented with 1.5% BSA (160069, MP Biomedicals) and 1 mg ml^−1^ collagenase type 1 (LS004194, Worthington Biochemical) on a shaking water bath (at 135 rpm at 37 °C) for 30–45 min. The digested tissue was then filtered through a 100-µm mesh strainer, mixed with an equal volume of DMEM supplemented with 10% FBS to inactivate collagenase, and subsequently centrifuged at 400*g* for 5 min to separate the SVF and adipocytes. The SVF was washed, resuspended in DMEM supplemented with 10% FBS, and transferred to culture plates for further experiments. The floating adipocytes were washed three times with culture medium (DMEM-F12 supplemented with 10% FBS and 1% penicillin–streptomycin) at room temperature. During each wash, adipocytes were allowed to float for 3 min before removing the infranatant with a syringe and needle. After the final wash, the isolated adipocytes were suspended in additional medium and embedded in Matrigel (356231, Corning) for imaging.

### Cell culture

The SVF was isolated from iWAT, C3H10T1/2 (CCL-226, American Type Culture Collection) cells, and 293T (CRL-3216, American Type Culture Collection) cells were maintained in DMEM supplemented with 10% FBS. To induce adipocyte differentiation, confluent SVF or C3H10T1/2 cells were cultured with media containing an adipogenic cocktail consisting of 2.5 μM or 1 μM dexamethasone (D4902, Sigma), 5 µg ml^−1^ insulin (I0516, Sigma), 500 μM isobutylmethylxanthine (I5879, Sigma) and 1 μM rosiglitazone (R2408, Sigma) for 2–3 days. Subsequently, the cells were switched to maintenance medium (culture medium supplemented with 5 µg ml^−1^ insulin). For *Lats1/Lats2* deletion in mature adipocytes differentiated from iAKO SVF, cells were treated with 1 µM 4-hydroxytamoxifen (H7904, Sigma) for 10 days, starting at 7 days after adipocyte differentiation. C3H10T1/2 cells transduced with TAZ4SA-ER^T2^ retroviruses containing a puromycin-resistance cassette were subjected to puromycin (2 µg ml^−1^) selection to establish tamoxifen-inducible TAZ4SA cells. Next, 1 µM 4-hydroxytamoxifen (H6278, Sigma) was treated for 24 h to activate TAZ4SA-ER^T2^. For adenoviral expression, cells were infected with adenoviruses encoding TAZ4SA or GFP as described previously^51^. For pharmacological inhibition or activation of YAP/TAZ, C3H10T1/2 day-10 adipocytes were treated with 5 μM verteporfin (SML0534, Sigma) for 12 h or C3H10T1/2 day-8 adipocytes were treated with 2 μM lysophosphatidic acid (L7260, Sigma) for 2 h after 12 h of serum starvation. To modulate YAP/TAZ pharmacologically, C3H10T1/2 cells at 8 or 10 days after adipocyte differentiation were treated with either 2 μM lysophosphatidic acid (L7260, Sigma) for 2 h following 12 h of serum starvation or 5 μM verteporfin (SML0534, Sigma) for 12 h, respectively, with ethanol as the vehicle for verteporfin and DMSO for lysophosphatidic acid.

### Bulk RNA-seq and analysis

RNA libraries were prepared using the TruSeq stranded total RNA library kit (Illumina). Sequencing was performed on an Illumina NovaSeq 6000 with 100 bp paired-end reads. Raw sequencing reads were mapped to the mm10 transcriptome using ‘salmon’ (v1.9.0, parameters: --numBootstraps 30 --libType A --seqBias --gcBias --reduceGCMemory)^52^, and then summarized at the gene level using the R package ‘tximeta’ (v1.12.4)^53^. Differentially expressed gene analysis was performed with the R package ‘DEseq2’ (v1.34.0)^54^. Pathway enrichment analysis was performed with GSEA software (4.1.0)^55^, using KEGG pathway gene sets (C2) and oncogenic signature gene sets (C6) from the Molecular Signature Database^55^.

### Single-cell RNA-seq

SVF cells were isolated from the iWAT of 4-week-old *Lats1*^fl/fl^; *Lats2*^fl/fl^; *Rosa-LSL-tdTomato* (Con) and *Adipoq-Cre; Lats1*^fl/fl^; *Lats2*^fl/fl^; *Rosa-LSL-tdTomato* (AKO) mice. The SVF pooled from 3–5 mice per genotype was sorted using a flow cytometer (BD Aria II) to exclude CD45^+^ leucocytes as previously described^21^. Briefly, the SVF underwent RBC lysis (11814389001, Roche) for 3 min, before washing with HBSS/3% BSA. Subsequently, cells were incubated with APC-CD45 antibody (559864, BD Pharmingen) at a 1:200 ratio diluted in HBSS/3% BSA for 20 min on ice, washed with HBSS/3% BSA and resuspended in FACS buffer (PBS/0.5% BSA). CD45^−^/tdT^+^ cells from the AKO SVF and CD45^−^/tdT^−^ cells from the control SVF were sorted for single-cell RNA-seq. In total, 5,000 single-sorted cells were targeted and processed for single-cell library generation using 10x Chromium Single Cell 3′ reagent kit v3 (10x Genomics), following the manufacturer’s protocols. Single-cell libraries were sequenced on the HiSeq-X platform (Illumina).

### Single-cell RNA-seq data analysis

Single-cell RNA-seq data were aligned and quantified using the STAR v2.7.6a. A STAR genome index was generated for the GRCm38 mouse genome assembly with Gencode M23 annotations. STARsolo was run with the parameters: --soloType CB_UMI_Simple–soloUMIlen 12 --soloBarcodeReadLength 0 --soloStrand Forward --soloUMIfiltering MultiGeneUMI --soloCellFilter CellRanger2.2 3000 0.99 10 --soloFeatures Gene GeneFull; all other parameters used default values. Low-quality cell barcodes were removed based on unique molecular modifier (UMI) counts of less than 2,000 and less than 500 genes detected, and more than 7,000 genes detected and high mitochondrial content. The downstream analysis included data normalization, highly variable gene detection, log transformation, principal component analysis, neighbourhood graph generation and Leiden graph-based clustering and batch integration (Harmony), which was done by Python package scanpy (v1.8.2) using default parameters.

### FACS

SVF cell preparations were incubated with fluorescent-conjugated primary antibodies at a 1:200 ratio for 30 min on ice. After washing with PBS, cells were sorted using a FACS Aria III instrument (BD Biosciences). APC-CD31 antibody (102410, BioLegend) and APC-CD45 antibody (103112, BioLegend) were used to exclude endothelial and immune cells. PeCy7-DPP4 antibody (137809, BioLegend) and PeCy7-ICAM1 antibody (116122, BioLegend) were used to sort adipocyte progenitor cells.

### Western blot analysis

Adipose tissues were homogenized in RIPA buffer supplemented with protease/phosphatase inhibitor (P3300, GenDEPOT), resolved by Tris-glycine SDS–PAGE, and subjected to standard ECL immunoblotting. The following antibodies were used for western blot analysis at a 1:1,000 dilution: LATS2 (5888S, Cell Signaling Technology), YAP/TAZ (8418S, Cell Signaling Technology), Phospho-YAP (4911S, Cell Signaling Technology), vinculin (13901S, Cell Signaling Technology), LATS1 (A300-477A, Bethyl) and AMOTL2 (ab221131, Abcam).

### Mouse metabolic phenotyping

Glucose tolerance tests were performed by intraperitoneal injection of glucose (2 g per kg body weight) in mice following an overnight fast. Blood was collected from the tail vein at the indicated timepoints, and blood glucose levels were measured with a glucometer (Allmedicus). Fat mass and lean mass were determined with a time-domain nuclear magnetic resonance spectrometer (Minispec LF50, Bruker Biospin). For indirect calorimetry and activity measurement, mice were individually housed in the Oxymax-CLAMS chamber (Columbus Instruments) and analysed for oxygen consumption, carbon dioxide production and locomotor activity over 5 consecutive days. The RER was calculated as *V*CO_2_/*V*O_2_, and energy expenditure was calculated as (3.815 + 1.232 × RER) × *V*O_2_. Serum free fatty acid and triglyceride levels were measured with a Cobas 8000 modular analyser (Roche). Serum leptin (22-LEPMS-E01, ALPCO) and insulin (80-INSMSU-E10, ALPCO) levels were determined through ELISAs. Serum ALT was measured with the IDEXX VetTest chemistry analyser (98-24010-US, IDEXX VetTest).

### Tissue histology

Briefly, tissue samples were fixed using zinc formalin fixative (Z2902, Sigma), dehydrated in ethanol, cleared with xylene, embedded in paraffin and sectioned at a thickness of 4 µm. The sections were deparaffinized, rehydrated, stained with Mayer’s haematoxylin (HHS32, Sigma-Aldrich) and eosin Y (HT110280, Sigma-Aldrich), dehydrated and mounted in DPX mounting medium (44581, Sigma-Aldrich).

### Fluorescence imaging of adipose tissue and cells

For whole-mount fluorescence microscopy of adipose tissue, fascia was carefully removed from inguinal fat pads under a dissecting microscope and the tissue was then fixed for 1 h at room temperature with 1% paraformaldehyde in PBS and permeabilized for 1 h with 0.3% Triton X-100 in PBS (PBST) before incubation for 30 min at room temperature with Hoechst 33342 (H3570, Invitrogen) at a dilution of 1:1,000 and boron dipyrromethene (BODIPY; D3822, Invitrogen) at a dilution of 1:2,000 in PBST. The tissue was washed several times with PBST, mounted in fluorescence mounting medium (S3023, Dako) and imaged with a confocal microscope (LSM880, Carl Zeiss). For Oil Red O staining of lipid droplets, cells were fixed with 4% formaldehyde, stained with Oil Red O solution (O1516, Sigma) for 20 min, and washed with PBS. Fluorescence imaging of cells was performed after staining with Hoechst 33342 (ab228551, Abcam) and BODIPY 493/503 (D3922, Thermo Fisher Scientific). Lipid droplet accumulation was assessed at 6 days after application of the adipogenic cocktail to induce adipocyte differentiation. The cells were imaged using a CQ1 microscope (Yokogawa).

### Immunofluorescence analysis

Formalin-fixed paraffin sections were deparaffinized, rehydrated and immersed in PBS. Antigen retrieval was performed with citric acid (0.01 M, pH 6.0), and non-specific sites of the sections were blocked by incubation with PBS containing 10% donkey serum and 0.2% Triton X-100 for 1 h. The sections were incubated overnight at 4 °C with primary antibodies diluted in PBS containing 0.2% Triton X-100. Immune complexes were detected by incubation for 1 h at room temperature with Alexa Fluor 488-, Alexa Fluor 594- or Alexa Fluor 647-conjugated secondary antibodies at a dilution of 1:500 in PBS. The sections were then washed three times for 5 min with PBS, exposed to DAPI (D9542, Sigma) for 5 min to stain nuclei, washed briefly with PBS, mounted with ProLong Gold antifade reagent (P36930, Invitrogen) and sealed with a cover glass (0101172 or 0101192, Marienfeld). Primary antibodies were used at dilutions of 1:500 for red fluorescent protein (600401379, Rockland, or AB8181-200, Sicgen) and Ki67 (ab16667, Abcam), 1:200 for PPARG (sc-7273, Santa Cruz Biotechnology), PDGFRA (AF1062, R&D Systems) and YAP/TAZ (8418S, Cell Signaling).

### RT–qPCR analysis

Total RNA was isolated from tissue with the use of TRIzol (15596018, Invitrogen) and from cultured cells with the use of an RNeasy Kit (74106, Qiagen). RNA was subjected to reverse transcription with the use of a High-Capacity cDNA Reverse Transcription Kit (4368814, Applied Biosystems), and the resulting cDNA was subjected to real-time PCR analysis with Fast SYBR Green Master Mix (4385612, Applied Biosystems) in a ViiA 7 real-time PCR system (Applied Biosystems). Relative gene expression was calculated by the delta delta Ct (ΔΔC_T_) method and was normalized by *L32* or *36b4* gene expression. PCR primer sequences are provided in Supplementary Table 1.

### CRISPR–Cas9-mediated genome editing

Guide RNA primers (Supplementary Table 1) targeting the TEAD-binding site of the mouse *Lep* enhancer were annealed and ligated to the lentiCRISPR v2 hygro-vector (98291, Addgene)^56^. C3H10T1/2 cells were infected with the lentivirus in the presence of Polybrene (H9268, Sigma) at 6 μg ml^−1^ for 24 h and then subjected to selection with hygromycin (400 μg ml^−1^). Clones were isolated and screened for successful ablation of the target region by PCR analysis of genomic DNA and sequencing.

### Luciferase reporter assay

An ~1.2-kb genomic fragment containing the TAZ-binding region located 28 kb upstream of the *Lep* transcription start site (Supplementary Table 1) was cloned into the pGL3 luciferase reporter plasmid (E1751, Promega) using the In-Fusion Cloning Kit (638920, Takara). Luciferase activity was measured in 293T cells 24 h after transfection with luciferase reporter plasmids, along with an expression vector for YAP5SA (constitutively active YAP) or YAP5SA_94A (YAP5SA lacking an intact TEAD-interacting domain) and a plasmid encoding *Renilla* luciferase (E1960, Dual-Luciferase Reporter Assay System, Promega) as a control for transfection efficiency.

### ChIP

ChIP was performed as previously described^57^, with slight modifications. Briefly, cells were cross-linked with 1.5% formaldehyde for 10 min, and nuclear extracts were sonicated. Immunoprecipitation was performed overnight at 4 °C using antibodies diluted to 1:100 for TAZ (560235, BD Biosciences), YAP (2F12, YF-MA11283, AbFrontier) or mouse IgG (sc-2025, Santa Cruz), all prebound to Dynabeads Protein G (Thermo Fisher Scientific). Antibody-bound DNA fragments were purified using the MinElute PCR Purification Kit (28006, Qiagen) and subjected to sequencing library preparation or qPCR analysis of the *Lep* enhancer region or *B2m* gene (non-specific control).

### ChIP–seq and computational analysis

Sequencing libraries were constructed with the use of a TruSeq ChIP–seq Library Prep Kit (Illumina) and sequenced with an Illumina NovaSeq 6000 system. Sequenced reads were trimmed and aligned to mouse genome mm10 with Bowtie 2. After removal of duplicated reads and peak calling, common peaks between two replicates were used for genomic feature annotation and de novo motif analysis with ChIPseeker and HOMER, respectively. For H3K27ac and H3K4me1 ChIP–seq data (GSE74189)^33^, mm9 genome-mapped BigWig files were converted to mm10 using CrossMap and visualized on the UCSC genome browser.

### Stable isotope tracer infusion

In vivo mice infusion experiments and metabolic flux analysis were performed by Myocare. Mice deprived of food for 6 h were subjected to a primed constant infusion of [1-^13^C]bicarbonate (372382, Sigma) (prime, 12.75 nM per gram of body weight; rate, 0.15 nM per gram of body weight per minute) for 60 min, followed by infusion of [U-^13^C_16_]palmitate (CLM-3943) at a rate of 1.85 nM per gram of body weight per minute, [1,1,2,3,3-d_5_]glycerol (DLM-1129) at a rate of 2.5 nM per gram of body weight per minute for the next 120 min, via the jugular vein with the use of a dual-channel swivel and a closed metabolic system. Expired air and blood samples were collected at 0, 50, 55, 60, 140, 145 and 150 min and at 160, 170 and 180 min, respectively, after the onset of [1-^13^C] bicarbonate infusion. Tissues were then rapidly excised, frozen and stored at −80 °C until analysis.

### Measurement of stable isotopic enrichment by mass spectrometry

For analysis of metabolites, plasma and the soluble fraction of tissue homogenates were dried and derivatized as previously described^58^. Enrichment of metabolites was analysed by gas chromatography (GC) and mass spectrometry (5977B, 8890, Agilent). Exhaled CO_2_ samples were analysed to determine ^13^C enrichment with a trace gas analyser–isotope ratio mass spectrometry (IRMS) system (Isoprime) and with a trace gas preconcentration unit as previously described^59^. In brief, CO_2_ gas injected with a gas-tight syringe was cryogenically concentrated in glass-lined cryofocusing traps immersed in liquid nitrogen and was separated on a 30-m gas chromatography capillary column filled with Poraplot Q (ChromPack, Varian). The analytes of the sample and reference gases were then introduced into the IRMS instrument to measure the abundance of ions with mass/charge (*m/z*) ratios of 44, 45 and 46 for CO_2_.

### Calculation of metabolite kinetics

For determination of the rate of appearance (*R*a) of tracee, respective tracer infusion rates (*F*) were divided by isotopic enrichment at the plateau, which was expressed as mole percent excess. In a steady state, in which tracee pool size is constant, *R*a is equal to the rate of disappearance (*R*d)^60^. The rate of palmitate oxidation is typically calculated as the product of palmitate uptake (that is, *R*d of palmitate) and the fraction of *R*d for palmitate that was oxidated, as previously described^59^. In the present study, the rate of palmitate oxidation was calculated with a modified equation, in which *V*CO_2_ was replaced with the product of *R*a for CO_2_ and *C* (recovery factor for ^13^CO_2_ retention), given that *V*CO_2_ is equal to this product. The *R*a for CO_2_ was calculated by dividing *F* by enrichment of CO_2_ (ECO_2_) of [1-^13^C] bicarbonate and then multiplying the quotient by ECO_2_ of [U-^13^C_16_] palmitate. The product was then divided by the enrichment of plasma palmitate. Given that the complete oxidation of 1 mole of [U-^13^C_16_] palmitate produces 16 moles of ^13^CO_2_, ^13^CO_2_ enrichment must be divided by the number of labelled atoms (*n* = 16). The contribution of palmitate to tricarboxylic acid cycle flux in various tissues was measured on the basis of the abundance of ions with *m/z* ratios 459, 460, 461, 462, 463, 464 and 465 for citrate (M + 0 to M + 6)^58^. The fractional contribution of palmitate to the tricarboxylic acid cycle was quantified from total citrate enrichment (sum of M + 1 to M + 6) normalized by plasma palmitate enrichment.

### Systems genetics analysis

The UK Biobank (UKBB) resource under application number 48020 was used. The phenotypic data of waist circumference (Data-Field 48, *n* = 500,203), hip circumference (Data-Field 49, *n* = 500,144), standing height (Data-Field 50, *n* = 499,825), glycated haemoglobin (HbA1c; Data-Field 30750, *n* = 466,376), weight (Data-Field 21002, *n* = 499,589) and glucose (Data-Field 30740, *n* = 429,452) were first downloaded from the UKBB^61^. WHR and A body shape index (ABSI) were calculated according to the previously described methods^62^. In total, 200,030 individuals with WGS data^63^ in the UKBB were selected and then the population of European descent (including 173,118 individuals with WHR, 171,998 individuals with ABSI, 165,547 individuals with HbA1c, 172,940 individuals with weight and 151,568 individuals with glucose) was extracted for further GWAS analyses. WGS data provided by the UKBB and used for GWAS were processed starting from pVCF files generated by GraphTyper Variant Calling^64^. Based on the WGS data, REGENIE step 1 was applied to estimate the population structure of each phenotypic trait, and then we used REGENIE step 2 to examine the genetic variant–phenotype associations for each phenotype. The following covariates were included in our model: the first ten genetic principal components, age, sex and age–sex interaction.

### Statistics and reproducibility

All quantitative data are presented as means ± s.e.m. All data were analysed with the two-tailed unpaired *t*-test for comparisons between two groups, except for Figs. 5e–g and 6e,f, which were analysed by one-way analysis of variance, and Fig. 6l, which was analysed by two-way analysis of variance, followed by Tukey’s post hoc test for comparisons among more than two groups. Statistical analysis was performed with GraphPad Prism (v8.03) software. Data shown in Figs. 1e–g,l and 2b,c, Extended Data Figs. 1f and 4a,c and Supplementary Fig. 1a,b were repeated more than three times with similar results.

### Reporting summary

Further information on research design is available in the Nature Portfolio Reporting Summary linked to this article.

### Supplementary information

Supplementary Information Supplementary Figs. 1–4 and Table 1.

Reporting Summary

Supplementary Data Source data for Supplementary Figs. 1–4.

### Source data

Source Data Figs. 1–6 and Extended Data Figs. 1–10 Statistical source data.

Source Data Extended Data Fig. 9 Full-length, unprocessed blots.

## Data Availability

RNA-seq, single-cell RNA-seq and ChIP–seq data generated during the current study are available under the accession codes GSE203417 and GSE261825. Source data are provided with this paper.
