# Supplementary material for: Hippo–YAP/TAZ signalling coordinates adipose plasticity and energy balance by uncoupling leptin expression from fat mass
Source: Nat Metab. 2024 May 29;6(5):847–60. doi: 10.1038/s42255-024-01045-4 (PMC11136666; doi:10.1038/s42255-024-01045-4)
Supplement: Supplementary file 1 — Supplementary Figs. 1–4 and Table 1. [file 42255_2024_1045_MOESM1_ESM.pdf]

nature metabolism

Supplementary information https://doi.org/10.1038/s42255-024-01045-4

Hippo–YAP/TAZ signalling coordinates adipose plasticity and energy balance by uncoupling leptin expression from fat mass

In the format provided by the authors and unedited

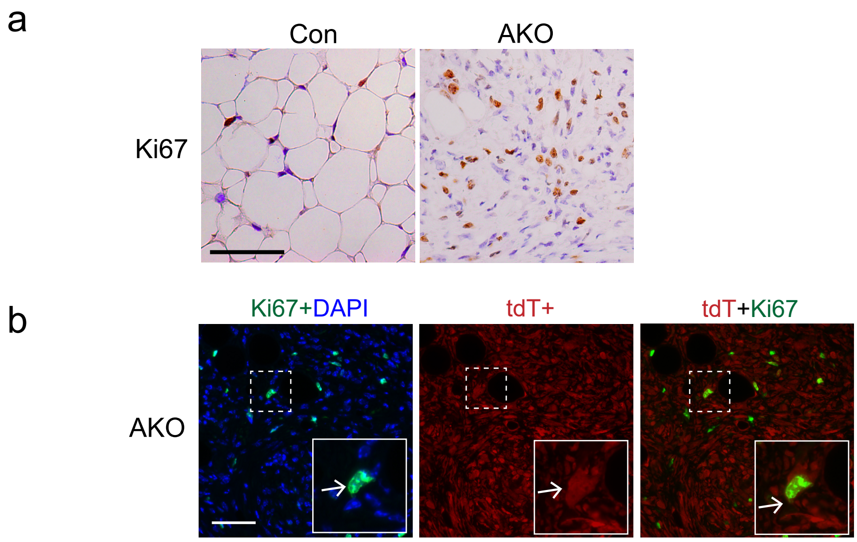

Supplementary Figure 1. Increased proliferative capacity of adipocyte-derived cells of AKO mice. iWAT of *Lats1$^{fl/fl}$*; *Lats2$^{fl/fl}$*; *Rosal-LSL-tdTomato*(control, Con) or *Adipoq-Cre*; *Lats1$^{fl/fl}$*; *Lats2$^{fl/fl}$*; *Rosa-LSL-tdTomato* (AKO) male mice at the age of 3-4 weeks was subjected to immunohistochemical staining of Ki-67 (**a**) as well as to immunofluorescence staining of Ki67 (green) and tdTomato (tdT, red) and nuclear staining with DAPI (blue) (**b**). The regions outlined by the dashed boxes in **b** are shown at higher magnification in the insets. The arrows indicate tdTomato and Ki67 colocalized nucleus. Scale bars = 50 µm

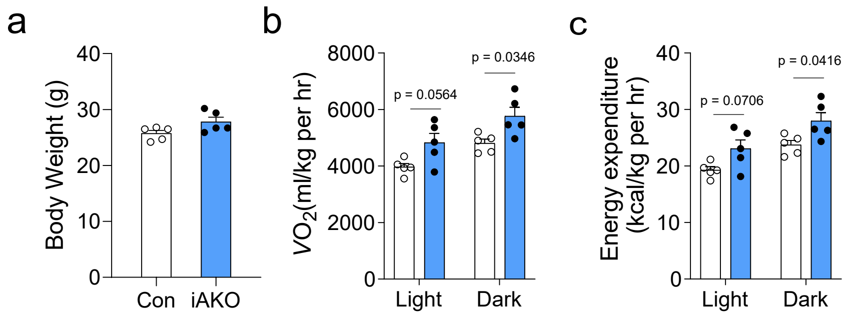

Supplementary Figure 2 (related to Figure 4c-d). Adipose-specific Lats1/2 deletion increases oxygen consumption and energy expenditure. a-b, *Lats1*$^{fl/fl}$; *Lats2*$^{fl/fl}$ (Con, control) and *Adipoq-CreER*; *Lats1*$^{fl/fl}$; *Lats2*$^{fl/fl}$ (iAKO) male mice at 8 to 10 weeks of age received tamoxifen injection for three times at every other day (100mg/kg). Mice were placed in a metabolic chamber for indirect calorimetry one day after the final tamoxifen injection, when there was no significant body weight difference between control and iAKO mice. Oxygen consumption rate (*V*O2) and energy expenditure were measured during the combined light or dark periods over 4 days. Body weight (**a**), oxygen consumption (*V*O2) (**b**), and energy expenditure (**c**) with lean mass normalization (n = 5 mice per group). Data expressed as mean ± s.e.m. Data were analyzed by two-tailed unpaired t test.

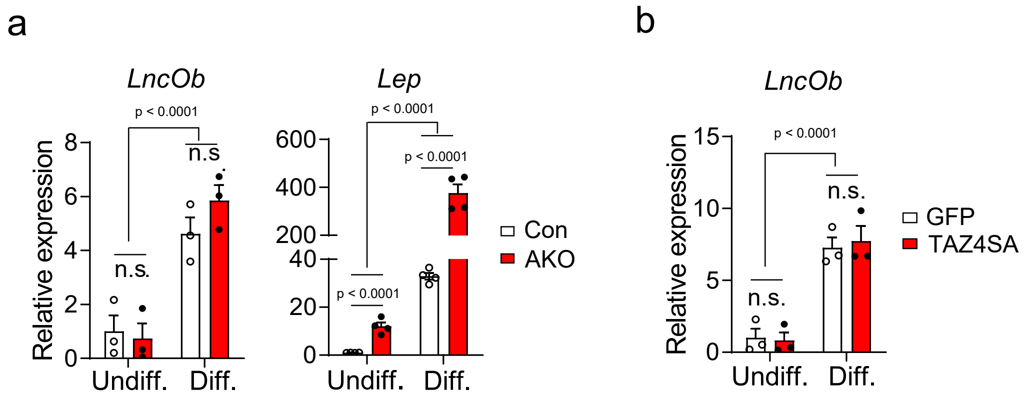

Supplementary Figure 3. *LncOb* gene expression is not associated with YAP/TAZ. a, RT-qPCR analysis of *LncOb* and *Lep* expression level in undifferentiated (Undiff.) or differentiated (Diff.) SVF cells from *Lats1*$^{fl/fl}$; *Lats2*$^{fl/fl}$ (control, Con) or *Adipoq-Cre*; *Lats1*$^{fl/fl}$; *Lats2*$^{fl/fl}$ (AKO) male and female mice (*LncOb* n= 3 per condition/genoype, *Lep* n=4 per condition/genotype). **b**, RT-qPCR analysis of *LncOb* expression in undifferentiated or differentiated C3H10T1/2 cells infected with an adenovirus encoding GFP or TAZ4SA as in **Figure 6a** (n= 3 per condition). Data expressed as mean ± s.e.m. Data were compared between Con and AKO or between GFP and TAZ4SA with the two-tailed unpaired t test, or between undifferentiated and differentiated samples by two-way ANOVA. n.s., not significant

```
(chr6:29,032,290-29,032,377)
   Lep enhancer CCTTCAGCAGCCTGGCTGCTCCCTCTGATCCTTGTTGGTGACATGAAGGGAATGCATGAAACTCTCTCTCTGGAGGCTCCTTCCAGC
ΔTEAD BE clone CCTTCAGCAGCCTGGCTGCTCCCTCTGATCCTTGTTGGTGA--------------ATGAAACTCTCTCTCTGGAGGCTCCTTCCAGC
      NS clone CCTTCAGCAGCCTGGCTGCTCCCTCTGATCCTTGTTGGTGACATGAAGGGAATGCATGAAACTCTCTCTCTGGAGGCTCCTTCCAGC
               *****************************************              ********************************
```

Supplementary Figure 4. Deletion of the TEAD binding element within the *Lep* enhancer region in C3H10T1/2 cells. Seqeunce alignment of the *Lep* enhancer region from the TEAD binding element (BE)-deleted (ΔTEAD BE) clone, generated using a TEAD BE-targeted gRNA, as well as a non-specific (NS) clone, generated using an NS gRNA. The TEAD BE seqeunces are highlighted in yellow, PAM sequences in red, and the indel (deleted sequences) are in bold.

Supplementary Table. Primer sequences

	**Forward**	**Reverse**
**RT-qPCR**		
Acc1	CAACGAGATTTCACTGTGGCT	TTCTGCATTGGCTTTAAGGTCT
Adipoq	GCACTGGCAAGTTCTACTGCAA	GTAGGTGAAGAGAACGGCCTTGT
Cepba	CAAAGCCAAGAAGTCGGTGGACAA	TCATTGTGACTGGTCAACTCCAGC
Cyr61	CTGCGCTAAACAACTCAACGA	GCAGATCCCTTTCAGAGCGG
Fabp4	CGCAGACGACAGGAAGGT	TTCCATCCCACTTCTGCAC
Fasn	AGAAGCCATGTGGGGAAGATT	AGCAGGGACAGGACAAGACAA
Lats1	TGGTGACTCTGGGGATAAAGAA	GGGAGTAACTCTGAATCCGAGAC
Lats2	GGACCCCAGGAATGAGCAG	CCCTCGTAGTTTGCACCACC
Lep	GGGCTGGAGGATGAACAAAG	CGCCGTGAGTAGGGTCTAA
LncOb	CTAGGCCCACATTCCTGATAC	GACTTTGCCTTCTTGGTTCTTG
Lpl	GCTGGTGGGAAATGATGTG	TGGACGTTGTCTAGGGGGTA
L32	GGCCTCTGGTGAAGCCCAAGATCG	CCTCTGGGTTTCCGCCAGTTTCGC
Pdgfra	AACGGAGGAGCTGCGGGGAA	CCCATAGCTCCTGAGACCTTCTCCT
Pdgfrb	AGGACAACCGTACCTTGGGTGACT	CAGTTCTGACACGTACCGGGTCTC
Plin1	GGTGTTACAGCGTGGAGAGTA	TCTGGAAGCACTCACAGGTC
Pparg	TCCAGCATTTCTGCTCCACA	ACAGACTCGGCACTCAATGG
Prdm16	CAGCACGGTGAAGCCATTC	GCGTGCATCCGCTTGTG
Pref1	AGTGCGAAACCTGGGTGTC	GCCTCCTTGTTGAAAGTGGTCA
Sca1	AGGAGGCAGCAGTTATTGTGG	CGTTGACCTTAGTACCCAGGA
Wisp2	CGCTGTGATGACGGTGGTTT	CCTGGCACCTGTATTCTCCTG
36b4	ATAACCCTGAAGTGCTCGACA	GGGAAGGTGTACTCAGTCTCC
**Luciferase construct**		
pGL3-mLep	GAGCTCGCTAGCCTCGAGGAGGGATC CTTTGGCCTTGATTG	GTACCGGATTGCCAAGCTTGAGGCATG TCAGAATGCAAGC
**ChIP**		
mLep-ChIP	GTGAGGTCACTGCATCCTGATTC	CCACTGAATGGCATTGCGTAC
mB2m-ChIP	GCCAAACCCTCTGTACTTCTCATTAC	GCACAGTGACAGACTTCAATTAGGC
**CRISPR KO**		
mLep-gRNA	CACCGATCCTTGTTGGTGACATGAA	AAACTTCATGTCACCAACAAGGATC
