# Supplementary material for: Hippo–YAP/TAZ signalling coordinates adipose plasticity and energy balance by uncoupling leptin expression from fat mass
Source: Nat Metab. 2024 May 29;6(5):847–60. doi: 10.1038/s42255-024-01045-4 (PMC11136666; doi:10.1038/s42255-024-01045-4)
Supplement: Supplementary file 2 — Reporting Summary [file 42255_2024_1045_MOESM2_ESM.pdf]

nature portfolio

Corresponding author(s): Jae Myoung Suh, Dae-Sik Lim

Last updated by author(s): Mar 12, 2024

Reporting Summary

Nature Portfolio wishes to improve the reproducibility of the work that we publish. This form provides structure for consistency and transparency in reporting. For further information on Nature Portfolio policies, see our Editorial Policies and the Editorial Policy Checklist.

Statistics

For all statistical analyses, confirm that the following items are present in the figure legend, table legend, main text, or Methods section.

n/a	Confirmed	
☐	☒	The exact sample size (*n*) for each experimental group/condition, given as a discrete number and unit of measurement
☐	☒	A statement on whether measurements were taken from distinct samples or whether the same sample was measured repeatedly
☐	☒	The statistical test(s) used AND whether they are one- or two-sided<br>*Only common tests should be described solely by name; describe more complex techniques in the Methods section.*
☐	☒	A description of all covariates tested
☒	☐	A description of any assumptions or corrections, such as tests of normality and adjustment for multiple comparisons
☐	☒	A full description of the statistical parameters including central tendency (e.g. means) or other basic estimates (e.g. regression coefficient) AND variation (e.g. standard deviation) or associated estimates of uncertainty (e.g. confidence intervals)
☐	☒	For null hypothesis testing, the test statistic (e.g. *F*, *t*, *r*) with confidence intervals, effect sizes, degrees of freedom and *P* value noted<br>*Give P values as exact values whenever suitable.*
☒	☐	For Bayesian analysis, information on the choice of priors and Markov chain Monte Carlo settings
☒	☐	For hierarchical and complex designs, identification of the appropriate level for tests and full reporting of outcomes
☒	☐	Estimates of effect sizes (e.g. Cohen's *d*, Pearson's *r*), indicating how they were calculated

Our web collection on statistics for biologists contains articles on many of the points above.

Software and code

Policy information about availability of computer code

Data collection	The following software were used for data collection:<br>Zen blue (v3.4) for immunofluorescence images<br>Quantstudio ViiA 7 Real-Time PCR system for CT values<br>BD Facs DIVA for FACS analysis
Data analysis	The following software were used for data analysis:<br>Zen 3.4 blue software for immunofluorescence images (Zeiss)<br>FastQC(v0.11.7), Trimmomatic (v0.38), Bowtie (v1.1.2), Picard MarkDuplicates(v1.1.8), MACS2 (v2.1.1) and ChIPseeker (v1.16.1) were used for ChIP-seq data analysis. No custom code was used.

For manuscripts utilizing custom algorithms or software that are central to the research but not yet described in published literature, software must be made available to editors and reviewers. We strongly encourage code deposition in a community repository (e.g. GitHub). See the Nature Portfolio guidelines for submitting code & software for further information.

Data

Policy information about availability of data

All manuscripts must include a data availability statement. This statement should provide the following information, where applicable:

- Accession codes, unique identifiers, or web links for publicly available datasets
- A description of any restrictions on data availability
- For clinical datasets or third party data, please ensure that the statement adheres to our policy

We have deposited our data on NCBI under GSE203417 (ChIP-seq, RNA-seq) and GSE261825 (scRNA-seq) accession code.
Following public data were used for our figures. Fig 6c (GSE74189), Fig 6d (GSE97972, ENCODE project, GeneHancer), Fig 6k (GSE138911). Extended Data Fig 1c (GSEA, Molecular Signatures Database)

Research involving human participants, their data, or biological material

Policy information about studies with human participants or human data. See also policy information about sex, gender (identity/presentation), and sexual orientation and race, ethnicity and racism.

Reporting on sex and gender	This study considered sex and age (40 to 74 years old) as covariates in the data analyses and these information was provided by the UK Biobank.
Reporting on race, ethnicity, or other socially relevant groupings	This study only considered European participants in the data analyses and this information was provided by the UKBB return dataset 2442
Population characteristics	This study considered sex and age (40 to 74 years old) as covariates in the data analyses and these information was provided by the UK Biobank.
Recruitment	Individuals are recruited by human UK Biobank.
Ethics oversight	We are allowed to use the UK Biobank Resource under Application Number 48020.

Note that full information on the approval of the study protocol must also be provided in the manuscript.

Field-specific reporting

Please select the one below that is the best fit for your research. If you are not sure, read the appropriate sections before making your selection.

☒ Life sciences ☐ Behavioural & social sciences ☐ Ecological, evolutionary & environmental sciences

For a reference copy of the document with all sections, see nature.com/documents/nr-reporting-summary-flat.pdf

Life sciences study design

All studies must disclose on these points even when the disclosure is negative.

Sample size	No statistical method was used to predetermine sample sizes. Due to the high reproducibility and consistency between cell cultures, in in vitro studies, it was predetermined that a sample size of at least n=3 would allow for adequate analysis to reach meaningful conclusions of the data. For our in vivo experiments, specific sample size for each experiment is indicated in the figure legends. These sample sizes were determined on the basis of previous experiments using similar methodologies and are sufficient to account for any biological or technical variabilities.
Data exclusions	For Fig 1i and 3g, one value was excluded due to a machine error. For Fig 4i, two values for soleus were excluded due to inadvertent loss of analyte during sample preparation. Also, one value for each iWAT and gWAT was excluded based on an outlier test (IQR method). For Ext Fig 2b, 2d, 2j, and 2k, we were unable to harvest adipose tissue from one mutant mouse due to its exceptionally small size, which fell below the threshold of visual identification.
Replication	All experiments conducted in this study were reproducible through repeated experiments. To verify the reproducibility of our findings, experiments were performed using at least three biological replicates.
Randomization	All mice were allocated in random. No bias in sample allocation was involved. For the remaining studies, randomization for different experimental groups was not relevant as they were performed on uniform biological material, i.e. cell lines procured from commercial sources
Blinding	The investigators were not blinded during data collection. Computational analysis was not performed blinded. Blinding is not relevant to the study as the output parameters are not subjective and therefore not subject to this form of bias. Blinding is also not relevant for bioinformatic analyses of large data sets as they are performed using computational algorithms.

Reporting for specific materials, systems and methods

We require information from authors about some types of materials, experimental systems and methods used in many studies. Here, indicate whether each material, system or method listed is relevant to your study. If you are not sure if a list item applies to your research, read the appropriate section before selecting a response.

Materials & experimental systems

n/a	Involved in the study
☐	☒ Antibodies
☐	☒ Eukaryotic cell lines
☒	☐ Palaeontology and archaeology
☐	☒ Animals and other organisms
☒	☐ Clinical data
☒	☐ Dual use research of concern
☒	☐ Plants

Methods

n/a	Involved in the study
☐	☒ ChIP-seq
☐	☒ Flow cytometry
☒	☐ MRI-based neuroimaging

Antibodies

Antibodies used

RFP (600401379, Rockland, 1:500), RFP (AB8181-200, Sicgen, 1:500), PPARG (sc-7273, Santa Cruz Biotechnology, 1;200), PDGFRA (AF1062, R&D Systems, 1:200), YAP/TAZ (8418S, Cell Signaling, 1:200 for IF, 1:1000 for WB), Ki67 (ab16667, Abcam, 1:500), TAZ (560235, BD Biosciences, 1:100), YAP (2F12, #YF-MA11283, AbFrontier, 1:100), mouse lgG (sc-2025, Santa Cruz, 1:100), APC-CD31 (102410, Biolegend, 1:200), APC-CD45 (103112, Biolegend, 1:200), PeCy7-DPP4 (137809, Biolegend, 1:200), PeCy7-ICAM1 antibody (116122, Biolegend, 1:200), LATS2 antibody (5888S, Cell Signal Tech, 1:000), Phospho-YAP (4911S, Cell Signal Tech, 1:000), Vinculin (13901S, Cell Signal Tech, 1:000), LATS1 (A300-477A, Bethyl, 1:1000) and AMOTL2 (ab221131, Abcam, 1:000).

Validation

Antibodies were chosen based on the available literature. All antibodies were provided by the manufacturer with validation data and citations.

-RFP antibody used in this manuscript was validated for immunofluorescence staining
(https://www.rockland.com/categories/primary-antibodies/rfp-antibody-pre-adsorbed-600-401-379/)

-RFP antibody used in this manuscript was validated for immunofluorescence staining
(http://www.sicgen.pt/product/tdtomato-polyclonal-antibody_1_135)

-PPARG antibody used in this manuscript was validated for immunofluorescence staining
(https://www.scbt.com/p/ppargamma-antibody-e-8?
gclid=CjwKCAjwpqCZBhAbEiwAa7pXeVjA3SXQy4RO4ad2Ios21Z_dTpIZHe0jMeqCkRQaIZAXUAUw8tpXBxoClHYQAvD_BwE)

-PDGFRA antibody used in this manuscript was validated for immunofluorescence staining (Refer to Citations)
(https://www.rndsystems.com/products/mouse-pdgf-ralpha-antibody_af1062#product-citations)

-YAP/TAZ antibody used in this manuscript was validated for immunofluorescence staining (Refer to Citations)
(https://www.cellsignal.com/products/primary-antibodies/yap-taz-d24e4-rabbit-mab/8418)

-Ki67 antibody used in this manuscript was validated for immunofluorescence staining
(https://www.abcam.com/Ki67-antibody-SP6-ab16667.html?gclsrc=aw.ds|
aw.ds&gclid=CjwKCAjwpqCZBhAbEiwAa7pXeZx9ORRTPJSo3tTDhPcFMHCOAAB2s49URlomzEjCJFmp9OQM_n0CrxoCszwQAvD_BwE)

-TAZ antibody used in this manuscript was validated for immunoprecipitation (Refer to Citations)
(https://www.bdbiosciences.com/ko-kr/products/reagents/microscopy-imaging-reagents/immunofluorescence-reagents/purified-mouse-anti-taz.560235)

-YAP antibody used in this manuscript was validated for immunoprecipitation
(https://www.embopress.org/doi/full/10.15252/embj.201695137)

-Mouse lgG used in this manuscript was validated as an isotype control immunoglobulin (Refer to Citations)
(https://www.scbt.com/p/normal-mouse-igg)

-APC-CD31 antibody used in this manuscript was validated for fluorescence-activated cell sorting analysis
(https://www.biolegend.com/en-us/products/apc-anti-mouse-cd31-antibody-118?GroupID=BLG2420)

-APC-CD45 antibody used in this manuscript was validated for fluorescence-activated cell sorting analysis
(https://www.biolegend.com/nl-be/products/apc-anti-mouse-cd45-antibody-97)

-PECy7-DPP4 antibody used in this manuscript was validated for fluorescence-activated cell sorting analysis
(https://www.biolegend.com/en-us/products/pe-cyanine7-anti-mouse-cd26-dpp-4-antibody-10368)

-PECy7-ICAM1 used in this manuscript was validated for fluorescence-activated cell sorting analysis

(https://www.biolegend.com/en-us/products/pe-cyanine7-anti-mouse-cd54-antibody-14759)

-LATS2 used in this manuscript was validated for western blot analysis
(https://www.sciencedirect.com/science/article/pii/S2211124722016497?via%3Dihub#sec4)

-YAP/TAZ used in this manuscript was validated for western blot analysis
(https://www.cellsignal.com/products/primary-antibodies/yap-taz-d24e4-rabbit-mab/8418)

-Phospho-YAP used in this manuscript was validated for western blot analysis
(https://www.cellsignal.com/products/primary-antibodies/phospho-yap-ser127-antibody/4911)

-Vinculin used in this manuscript was validated for western blot analysis
(https://www.cellsignal.com/products/primary-antibodies/vinculin-e1e9v-xp-rabbit-mab/13901)

-LATS1 used in this manuscript was validated for western blot analysis
(https://www.thermofisher.com/antibody/product/LATS1-Antibody-Polyclonal/A300-477A)

-AMOTL2 used in this manuscript was validated for western blot analysis
(https://www.abcam.com/products/primary-antibodies/amotl2-antibody-ab221131.html)

Eukaryotic cell lines

Policy information about cell lines and Sex and Gender in Research

Cell line source(s)	C3H10T1/2 and 293T cells were purchased from ATCC
Authentication	Cells were authentificated based on their morphology, growth condition and specific gene expression.
Mycoplasma contamination	All cell lines used in this study were tested negative for mycoplasma contamination.
Commonly misidentified lines (See ICLAC register)	No commonly misidentified cell lines were used in the study.

Animals and other research organisms

Policy information about studies involving animals; ARRIVE guidelines recommended for reporting animal research, and Sex and Gender in Research

Laboratory animals	Lats1fl/fl mice (024941, The Jackson Laboratory), Lats2fl/fl mice (PMID:23644383), Yap fl/fl mice (PMID:22028467), Taz fl/fl mice (PMID:23918388), Rosa26-LSL-tdTomato mice (007914, The Jackson Laboratory), Lepob/+ mice (000632, The Jackson Laboratory), Adipoq-Cre (010803, The Jackson Laboratory) and Adipoq-CreERT2 transgenic mice (024671, The Jackson Laboratory) were bred to generate mice used in this study. Their specific age is mentioned in the manuscript. Mice were housed under 12 light/12 dark cycle, temperatures of 22±2°C with 50±10% humidity.
Wild animals	No wild animals were captured for our study. Our mice samples were provided by approved mouse vendor.
Reporting on sex	Male mice were used except for the study related to Fig 2c, 2d, 6e and Fig 5 for which both male and females were used.
Field-collected samples	No wild animals were captured for our study. Our mice samples were provided by approved mouse vendor.
Ethics oversight	All protocols for mouse experiments were approved by the institutional animal care and use committee of the Korea Advanced Institute of Science and Technology

Note that full information on the approval of the study protocol must also be provided in the manuscript.

Plants

Seed stocks	N/A
Novel plant genotypes	N/A
Authentication	N/A

ChIP-seq

Data deposition

☒ Confirm that both raw and final processed data have been deposited in a public database such as GEO.

☒ Confirm that you have deposited or provided access to graph files (e.g. BED files) for the called peaks.

Data access links<br>*May remain private before publication.*	ChIP-seq data generated during the current study will be available after this manuscript is published as GSE203417
Files in database submission	Raw files:<br>JK_ChIP1_1.fastq.gz<br>JK_ChIP1_2.fastq.gz<br>JK_ChIP2_1.fastq.gz<br>JK_ChIP2_2.fastq.gz<br>JK_Input_1.fastq.gz<br>JK_Input_2.fastq.gz<br>Processed files:<br>JK_ChIP1_peaks.bed<br>JK_ChIP2_peaks.bed
Genome browser session<br>(e.g. UCSC)	N/A

Methodology

Replicates	For the experimental condition, 2 biological replicates were produced. Each replicates were processed and analyzed independently.
Sequencing depth	For every samples, 80 millions of paired-end reads (101bp) were generated.
Antibodies	TAZ (560235, BD Biosciences)
Peak calling parameters	Reads were trimmed using Trimmomatic and aligned to the UCSC mm10 genome using Bowtie. Duplicates were removed using Picard MarkDuplicates.<br>For each replicate, Peaks were called by MACS2 with input file and following parameters: -f BAMPE -q 0.05 -g mm --keep-dup 1 -B --SPMR --mfold 10 100 --call-summits
Data quality	FastQC was used to check the quality of raw and trimmed reads. ChIPseeker was used to TSS enrichment and distribuition of ChIP signals.
Software	FastQC(v0.11.7)<br>Trimmomatic (v0.38)<br>Bowtie (v1.1.2)<br>Picard MarkDuplicates(v1.1.8)<br>MACS2 (v2.1.1)<br>ChIPseeker (v1.16.1)

Flow Cytometry

Plots

Confirm that:

☒ The axis labels state the marker and fluorochrome used (e.g. CD4-FITC).

☒ The axis scales are clearly visible. Include numbers along axes only for bottom left plot of group (a 'group' is an analysis of identical markers).

☒ All plots are contour plots with outliers or pseudocolor plots.

☒ A numerical value for number of cells or percentage (with statistics) is provided.

Methodology

Sample preparation	Adipose tissues were minced and digested with collagenase buffer [0.1 M HEPES, 0.125 M NaCl, 5 mM KCl, 1.3 mM CaCl2, 5 mM glucose, 1.5% (w/v) bovine serum albumin, and 0.1% (w/v) collagenase I (49A18993, Worthington,) in a shaking water bath at 37 °C for 30–60 min. Dissociated cells were sequentially filtered through 100 µm and 40 µm nylon mesh strainers to remove undigested tissue and centrifuged at 400 × g for 5 min to obtain pelleted SVF. Cells were resuspended and incubated in RBC lysis buffer (1.7 M Tris, pH 7.65, and 0.16 M NH4Cl) for 3 min, filtered through a 35 µm nylon mesh strainer, and centrifuged at 400 × g for 5 min. The resulting SVF cell preparations were incubated with fluorescent conjugated primary antibodies at 1:200 ratio for 30 min on ice.
Instrument	After washing the cells several times with PBS, cells were sorted using a FACS Aria III instrument (BD Biosciences).

Software	BD FACS DIVA software
Cell population abundance	For single cell RNA-sequencing,<br>Contol iWAT SVF (FSC-A&SSC-A based selection 99.8%, Singlets 92.2%, CD45-&DAPI- 19.8%, tdTomato- 99.8%)<br>Mutant iWAT SVF (FSC-A&SSC-A based selection 98.3%, Singlets 85.8%, CD45-&DAPI- 31.3%, tdTomato+ 65.9%)<br><br>For adipocyte progenitor sorting,<br>Control DPP4 positive progenitor in iWAT SVF (FSC-A&SSC-A based selection 26.3%, Singlets 90.5%, DAPI- 99.5%, CD45-&CD31-44.5%, tdTomato- 99.9%, DPP4+ 62.9%)<br>Control ICAM1 positive progenitor in iWAT SVF (FSC-A&SSC-A based selection 26.8%, Singlets 90.4%, DAPI- 99.3%, CD45-&CD31-45.2%, tdTomato- 100%, ICAM1+ 45.1%)<br>Mut DPP4 positive progenitor in iWAT SVF (FSC-A&SSC-A based selection 32.8%, Singlets 88.2%, DAPI- 99.8%, CD45-&CD31-38.9%, tdTomato+ 72.1%, DPP4+ 23.5%)<br>Mut ICAM1 positive progenitor in iWAT SVF (FSC-A&SSC-A based selection 30.9%, Singlets 86.6%, DAPI- 100%, CD45-&CD31-41.3%, tdTomato+ 70.7%, ICAM1+ 46.2%)<br><br>For all conditions, FMO was used to select the right signal.
Gating strategy	Doublets were excluded based on forward scatter profiles. Live cells were selected using DAPI (Hoechst Blue). APC-CD31 antibody or APC-CD45 antibody were used to exclude endothelial or immune cells. tdTomato positive cells were selected based on PE signal with non-tdTomato cells as a negative control. DPP4 or ICAM1 positive cells were selected with PE-Cy7-DPP4 or PE-Cy7-ICAM1 antibodies.

☒ Tick this box to confirm that a figure exemplifying the gating strategy is provided in the Supplementary Information.
