# Supplementary figures and images for: Hippo–YAP/TAZ signalling coordinates adipose plasticity and energy balance by uncoupling leptin expression from fat mass
Source: Nat Metab. 2024 May 29;6(5):847–60. doi: 10.1038/s42255-024-01045-4 (PMC11136666; doi:10.1038/s42255-024-01045-4)

Full scan western image for Ext. Fig 9a

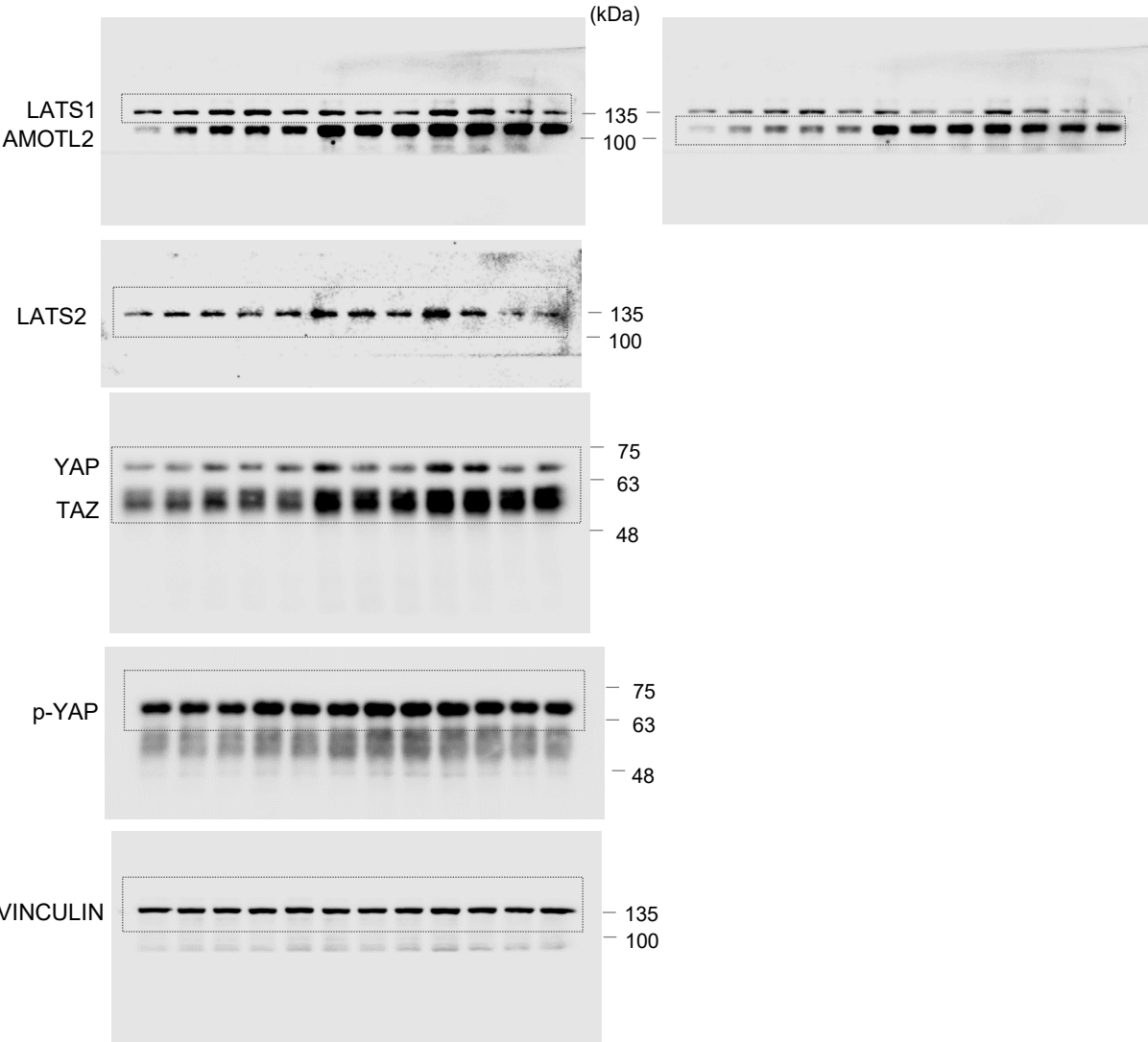

Full scan western image for Ext. Fig 9c

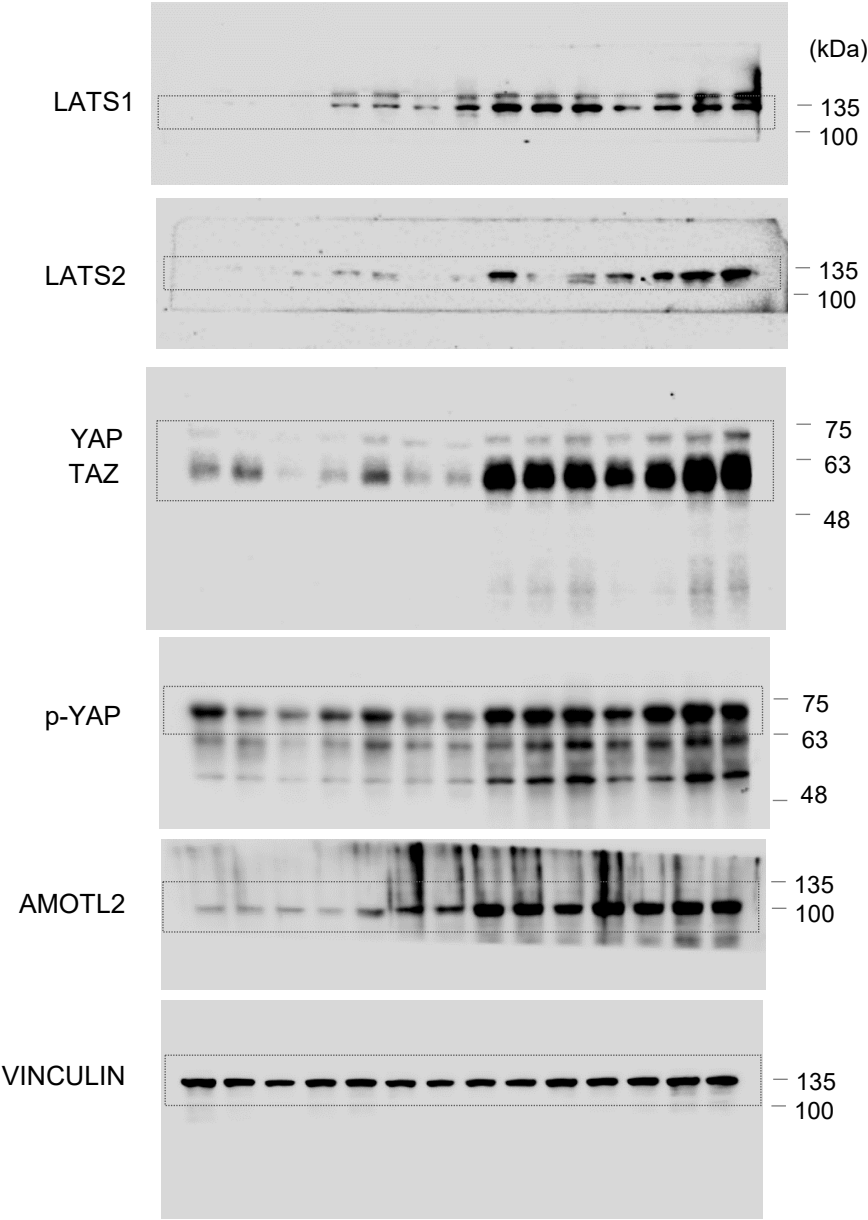

Supplement: Supplementary file 5 — Full-length, unprocessed blots. [file 42255_2024_1045_MOESM5_ESM.pdf]
